# Light-induced homolysis of copper(ii)-complexes – a perspective for photocatalysis

**DOI:** 10.1039/d3sc00388d

**Published:** 2023-04-06

**Authors:** Alexander Reichle, Oliver Reiser

**Affiliations:** a Institute of Organic Chemistry, University of Regensburg 93053 Regensburg Germany oliver.reiser@chemie.uni-regensburg.de

## Abstract

Over the past decade, photocatalysis has developed into a powerful strategy for the selective functionalization of molecules through radical intermediates. Besides the well-established iridium- or ruthenium-based photocatalysts, which ideally fulfill the requirements for a photocatalyst, such as long excited-state lifetimes and photostability, the shift towards earth-abundant metal-based photocatalysts has so far been less explored. The concept of light-induced homolysis (LIH) for generating radicals has recently gained significant interest as a new platform for inducing photoreactions with earth-abundant 3d-metal complexes despite only having excited-state lifetimes in the low nanosecond range or even below. Cu(ii)-complexes play a prominent role in exploiting this concept, which will be discussed by showcasing recent developments in organic synthesis with a view to identifying the future prospects of this growing field.

## Abstract 2

A new kid on the block: Cu(ii) offers unique possibilities in photocatalysis for generating and stabilizing radicals to promote challenging synthetic transformations.

## Graphical abstract

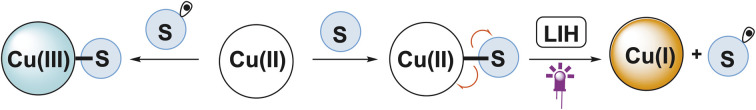

## 1. Introduction

Finding new ways to construct and break chemical bonds in simple operations is an essential task for synthetic chemists. The development of catalytic methods for the activation of small molecules is therefore of great interest for the utilization of resources and feedstocks in a sustainable way. In recent years, visible light photocatalysis has become a powerful tool enabling synthetic transformations.^1^ Being one of the latest disciplines in organic chemistry, the success and development of this field was proposed as early as 1912 by the Italian chemist G. Ciamican as a vision for truly green synthetic chemistry.^2^

**Fig. 1:**
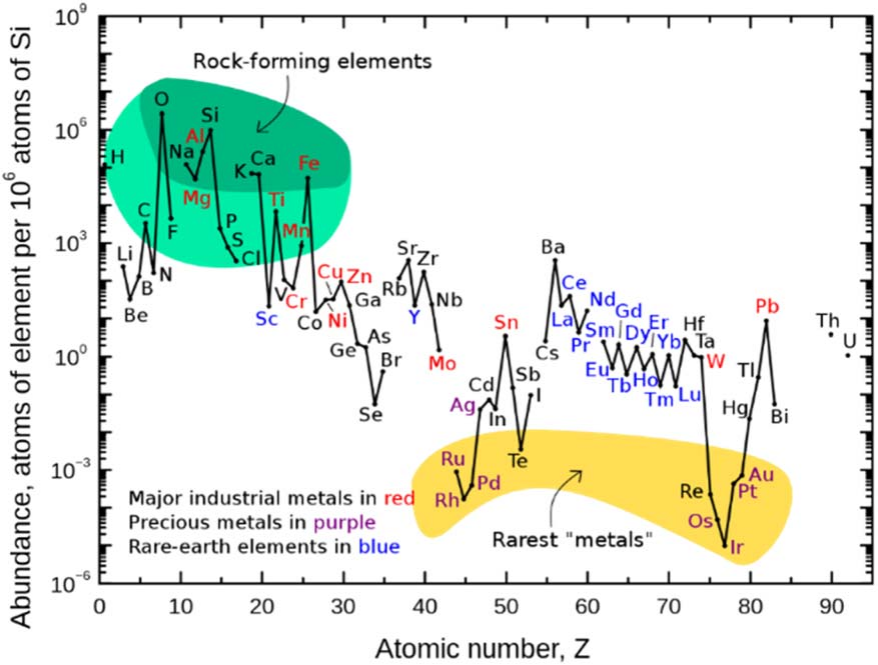
Abundance (atomic fraction) of the chemical elements in the Earth's upper continental crust as a function of atomic number. (Figure adapted from ref. ^5^, Creative Commons, public domain.)

Classical chemical bond formation occurs in the simplest case through an ionic pathway between electrophiles and nucleophiles. Despite the previous knowledge of methods to construct new chemical bonds using radical species, the lack of selectivity associated with the relatively harsh conditions necessary to generate such species hampered the development of sustainable radical pathways for organic synthesis^3^ despite impressive developments in this area.^4^ With the recent progress in harnessing light as an alternative to thermal treatment, a broad variety of radicals have become accessible offering new routes for the design of organic syntheses. As the principal photocatalysts that serve this purpose, precious heavy transition-metal polypyridinyl complexes based on iridium and ruthenium were arguably most widely employed. Such metal-based complexes possess excellent properties such as long excited-state lifetimes, high absorption coefficients in the visible range of the electromagnetic spectrum, and high oxidation and reduction potentials that are required to activate substrates *via* single electron transfer (SET) events. Nonetheless, they are disadvantageous in terms of sustainability and abundance.^5^ Therefore, shifting the focus from expensive Ir- and Ru-based photocatalysts to more abundant and environmentally benign 3d-metals like Cu, Fe, Co, Ni, or Mn has gained significant interest (Fig. 1).^6,7^

**Scheme 1:**
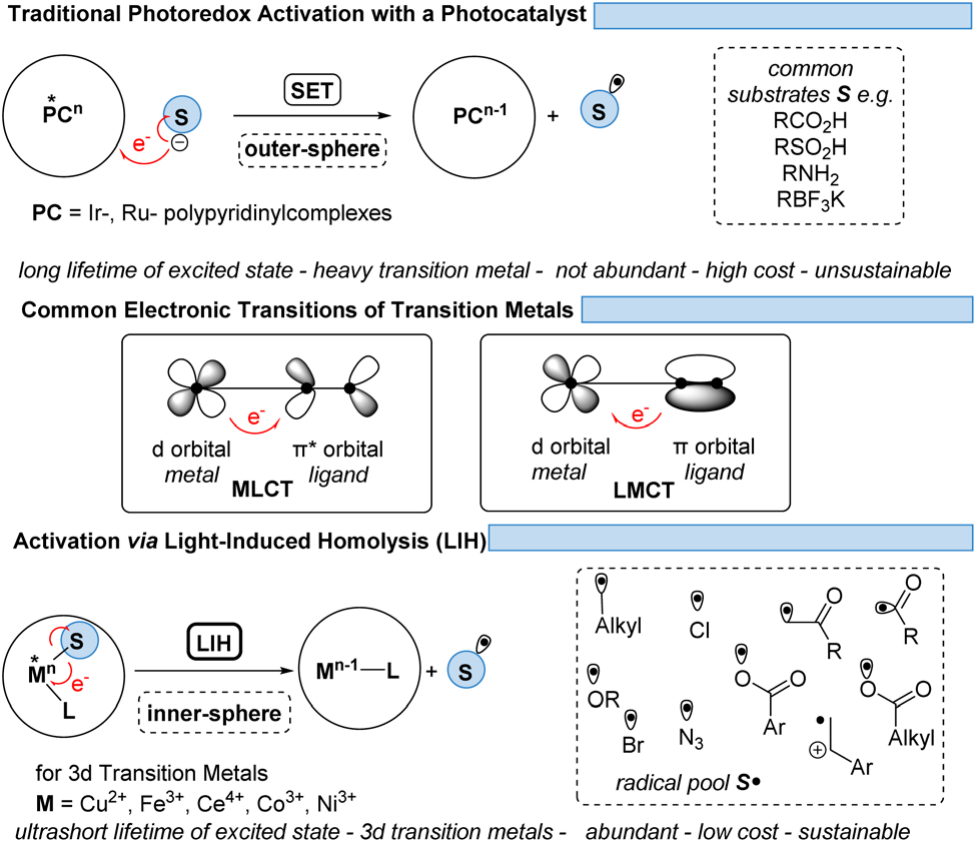
Traditional photoredox activation and the concept of LIH.

However, the generally ultrashort excited-state lifetimes of such metal complexes in the pico- or at best in the low nanosecond range^8^ make bimolecular processes *via* an outer-sphere mechanism unlikely. Nevertheless, homo- and heteroleptic copper(i)-complexes could be designed with excited-state lifetimes in the high nano- and even microsecond ranges. These could be successfully employed in synthetic transformations invoking single electron transfer (SET) from Cu(i)* to Cu(ii) along with the reduction of a substrate,^9,10^ echoing the chemical behavior known from Ru(ii)- or Ir(iii)-complexes in oxidative quenching cycles. In turn, the complementary reductive quenching cycles which are common for ruthenium (Ru(ii)* to Ru(i)) or iridium (Ir(iii)* to Ir(ii)) (Scheme 1) are scarce for copper(i) complexes (Cu(i)* to Cu(0)).^11,12^ Likewise, Cu(ii)-complexes that undergo photoinduced outer-sphere SET to give rise to Cu(i) and an oxidized substrate have not been exploited, reflecting the comparatively weak oxidation potential of Cu(ii) for such transitions. One alternative to utilizing Cu(ii) as a photocatalyst is the concept of light-induced homolysis (LIH), which is typically initiated by a ligand-to-metal charge transfer (LMCT) transition: upon excitation of a Cu(ii)–substrate complex, Cu(ii)–S, a homolytic dissociation to Cu(i) occurs and a substrate radical S˙ is formed which can be the starting point for subsequent synthetic transformations. The required Cu(ii)–substrate complex can be formed by a simple ligand exchange reaction with a nucleophilic substrate and a suitable Cu(ii)-precursor. Thus, the overall process is equivalent to the one-electron oxidation of the nucleophile. Such activation is advantageous because of the high chemoselectivity of the photochemical process since precoordination of the substrate is required. 3d metals are generally very efficient in coordinating diverse nucleophiles, with ample opportunity for tuning by appropriate choice of metal and ligands. Copper(ii) – being the focus of this review – has been the most commonly applied 3d-metal for this reaction mode,^13^ which is most likely also operating in thermal oxidation of substrates.^14^ Nevertheless, other 3d-metals such as Mn, Fe, Co and Ni as well as rare earth metals such as Ce have been successfully applied following this concept.^15,16^

Pioneering work of Kochi as early as the 1960s showed that metal complexes can undergo photolysis reactions, demonstrating in particular that under UV-irradiation CuCl_2_ forms CuCl + Cl˙.^17^ Nevertheless, the application of metal–substrate homolysis in synthetic transformations was not much explored until recently. There are several fundamental aspects involved, for which only now through the development of synthetic applications and insightful mechanistic studies a consistent picture is arising, which might serve as a toolbox for future developments. Questions to be answered are

• Which metal–(ligand)–substrate complexes can undergo light-induced homolysis reactions to generate substrate radicals?

• On what time scale does the homolysis reaction occur, *i.e.* are short excited-state lifetimes of 3d-metal complexes in the low nanosecond or even picosecond range amenable to initiating synthetic transformations?

• How efficient is the homolysis process, and specifically, does the possible recombination of the generated substrate radical with the reduced metal prevent the engagement of the former in further transformations?

• To make transformations catalytic, the reduced metal complex that forms upon homolysis needs to be reoxidized. If this is not possible, synthetic transformations might still be feasible and valuable, but stoichiometric amounts of the metal complexes have to be employed in such cases.

**Scheme 2:**
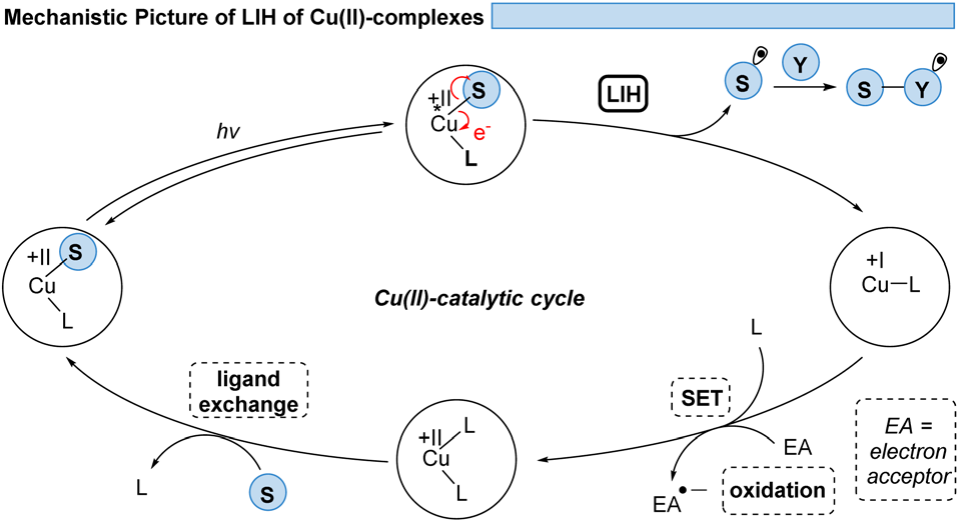
The general concept of LIH of Cu(ii)-intermediates.

Thus, for Cu(ii)-photocatalyzed transformations *via* LIH a blueprint (Scheme 2) can be proposed that can guide future developments: initially, a Cu(ii)–substrate complex must be formed, being achieved most commonly *via* ligand exchange of Cu(ii)L_2_ species with a nucleophilic substrate S. Upon irradiation this assembly undergoes light-induced homolysis to give rise to Cu(i) and a radical, S˙. The radical S˙ needs to then couple with a second substrate Y to initiate a productive synthetic transformation as opposed to the unproductive recombination with the reduced metal complex. The latter needs to be oxidized through a suitable electron acceptor to close the catalytic cycle. Notably, in many of the reported processes that involve LIH steps of Cu(ii)–substrate species, no external ligands are required. However, in such cases the reactions require near-UV-light (365 nm) or violet light (427 nm) irradiation because the LMCT transition of copper(ii)–substrate species requires higher energies to reach the dissociative states.^18^ So far, only a few Cu(ii)-intermediates are known to undergo light-induced homolysis (LIH). This review gives an overview of the reported transformations that use copper as a radical promoter through the LIH mechanism.

### 1.1 LIH of copper(ii) chloride-complexes

**Scheme 3:**
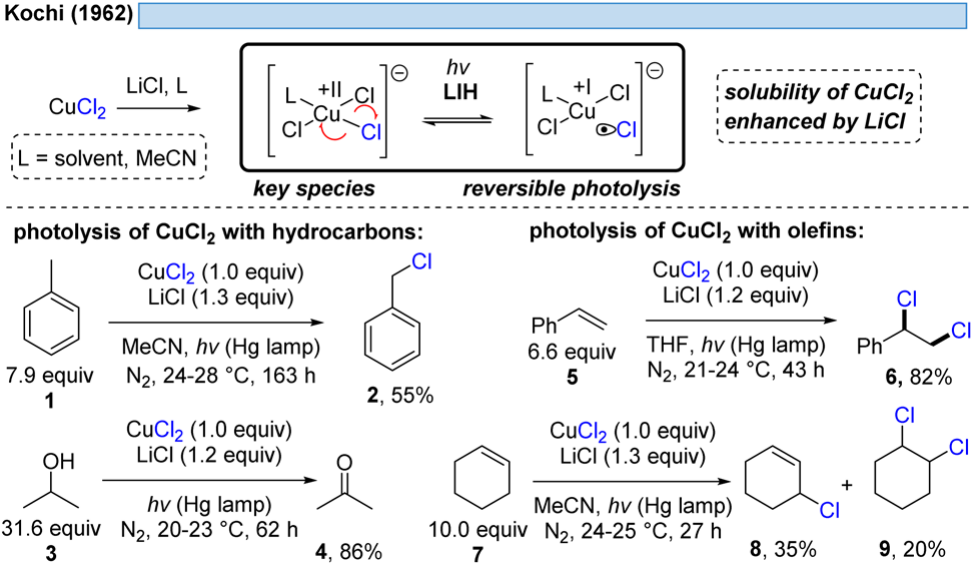
Pioneering work of Kochi in 1962: photolysis of cupric chloride (CuCl_2_).

The generation of chlorine radicals requires harsh conditions, given the high potential for the oxidation of Cl^−^ to Cl˙ (*E*_ox_ > +1.21 V *vs.* SCE),^19^ but can be nevertheless achieved using weakly oxidizing Cu(ii) (Cu(ii) → Cu(i), *E*_red_ = +0.50 V *vs.* SCE).^20,21^ In 1962, Kochi pioneered the field of light-induced homolysis by reporting the reversible homolysis of Cu(ii)Cl_2_ to Cu(i)Cl and a chlorine radical upon irradiation with a low-pressure mercury lamp.^17^ Adding lithium salts such as lithium chloride was found to be beneficial, with the reasoning being that the solubility of anhydrous cupric chloride (CuCl_2_) is increased, thus leading to enhanced activity. Only more than 50 years later did Mereshchenko and co-workers refine these observations by investigating the formation of several extremely labile copper(ii) chloride complexes with spectroscopic methods.^22^ They concluded that in acetonitrile (MeCN), the anionic complex [Cu^II^(MeCN)Cl_3_]^−^ is formed that undergoes reversible photolysis at a much higher rate (Scheme 3). This observation matched the investigations of Kochi: in solvents such as acetic acid and acetonitrile, almost no reaction was observable due to the low reactivity of the chlorine radicals towards these solvents, due to them being in competition with a fast rebound with Cu(i) to regenerate the initial Cu(ii)-complex. In contrast, solvents that bear structural motifs, such as secondary or tertiary C–H bonds or olefins, undergo transformations initiated by the chlorine radicals: with saturated hydrocarbons such as toluene (1), the formation of benzyl chloride (2) was observed. Likewise, the oxidation of isopropyl alcohol (3) to acetone (4) was achieved. From a mechanistic point of view, both reactions are initiated by hydrogen atom abstraction (HAT) of a chlorine radical. The photolysis of cupric chloride (CuCl_2_) in the presence of olefins, such as styrene (5) or cyclohexene (7), delivered the corresponding dichlorination products 6 and 8 as well as 9 in good to moderate yield, which is explained by the chlorine radical addition to the double bond. It should be noted that at this point CuCl_2_ is being employed in stoichiometric amounts, reflecting the problem of reoxidizing Cu(i) formed upon photolysis back to Cu(ii).

**Scheme 4:**
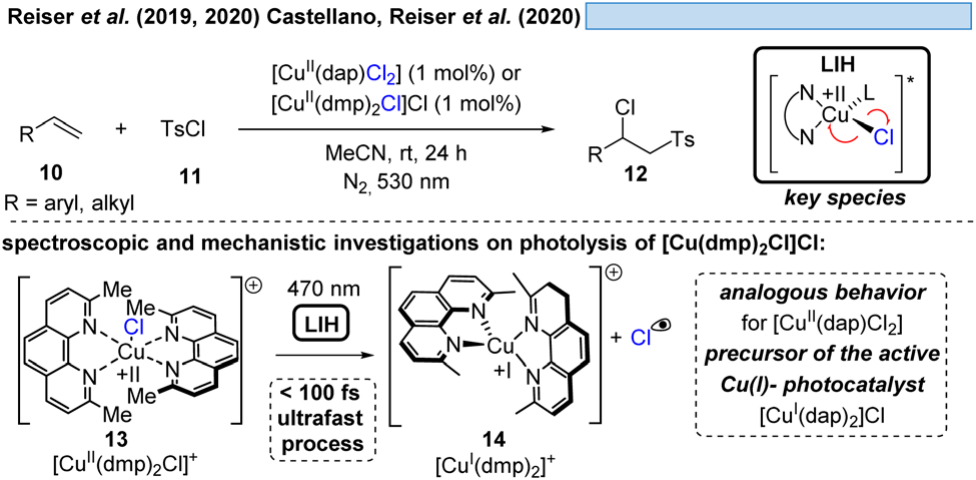
Cu(ii)-complexes as catalyst precursors: LIH of defined complexes.

In line with Kochi's proposal, it was found that copper(ii)-complexes such as [Cu^II^(dap)Cl_2_] (dap = 2,9-bis(4-methoxyphenyl)-1,10-phenanthroline) or [Cu^II^(dmp)_2_Cl]Cl (dmp = 2,9-dimethyl-1,10-phenanthroline) (13) can be employed in photocatalytic ATRA reactions as convenient and more economical precursors for the corresponding, catalytically active Cu(i)-complexes (Scheme 4). It was demonstrated that the Cu(ii)-complexes after photoexcitation undergo homolytic cleavage of the Cu–Cl bond to form *in situ* the catalytically active Cu(i)-species, which can promote a variety of ATRA reactions such as chlorosulfonylation, bromoalkylation or iodoperfluoroalkylation of alkenes^23^ following the mechanistic paradigm established for Cu(i)-photocatalyzed reactions.^9,10^ Taking complex 13 as a representative example, based on transient spectroscopy it was concluded that the homolysis of the Cu–Cl bond is an ultrafast process, occurring in less than 100 fs,^24^ which suggests that the photoinitiation of transformations *via* such bond homolyses is possible with metal complexes that have excited state lifetimes only in the picosecond range, *e.g.* opening a window to develop photocatalytic processes based on iron being the most abundant transition metal on earth.^7^

#### 1.1.1 LIH of CuCl_2_ – chlorine radical addition to alkenes/alkynes

**Scheme 5:**
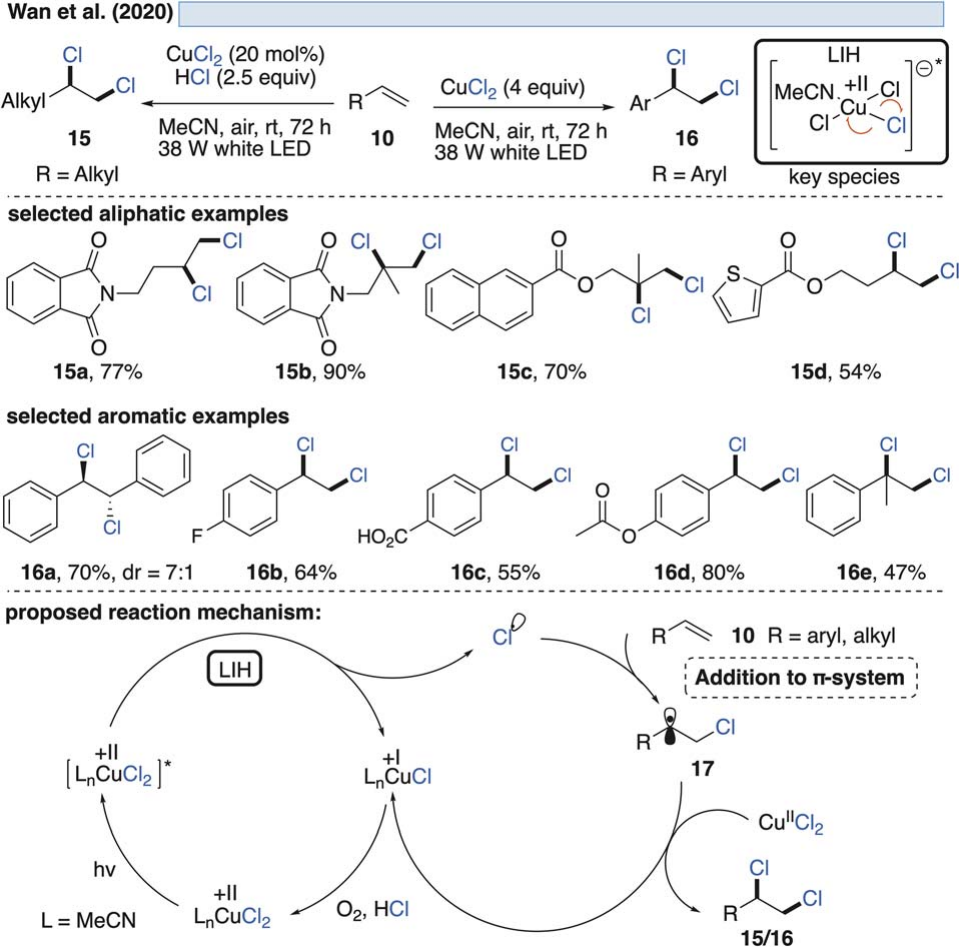
Vicinal dichlorination of activated and unactivated olefins.

Capitalizing on the discovery of Kochi, a practical procedure for the vicinal dichlorination of olefins 10 was described by Wan *et al.*^25^ (Scheme 5) to obtain products 15 and 16. For unactivated olefins, a combination of substoichiometric amounts of copper(ii) chloride (0.2 equiv.) and hydrochloric acid (2.5 equiv.) as an inexpensive and readily available chlorine source under aerobic conditions was found to be effective, giving rise to products of type 15. The reoxidation of Cu(i) that is formed upon homolysis of CuCl_2_, being critical to making the process catalytic, was proposed to take place in the presence of oxygen. Following this rationale, it is at first glance surprising that activated alkenes, *i.e.* vinyl arenes, required a large excess of CuCl_2_ (4.0 equiv.) to achieve the transformation. An explanation could be the competing trapping of benzylic radicals 17 (R = aryl) by oxygen, which was earlier demonstrated in the Cu(ii)-catalyzed azidoketonization (*vide infra*, Scheme 11).

**Scheme 6:**
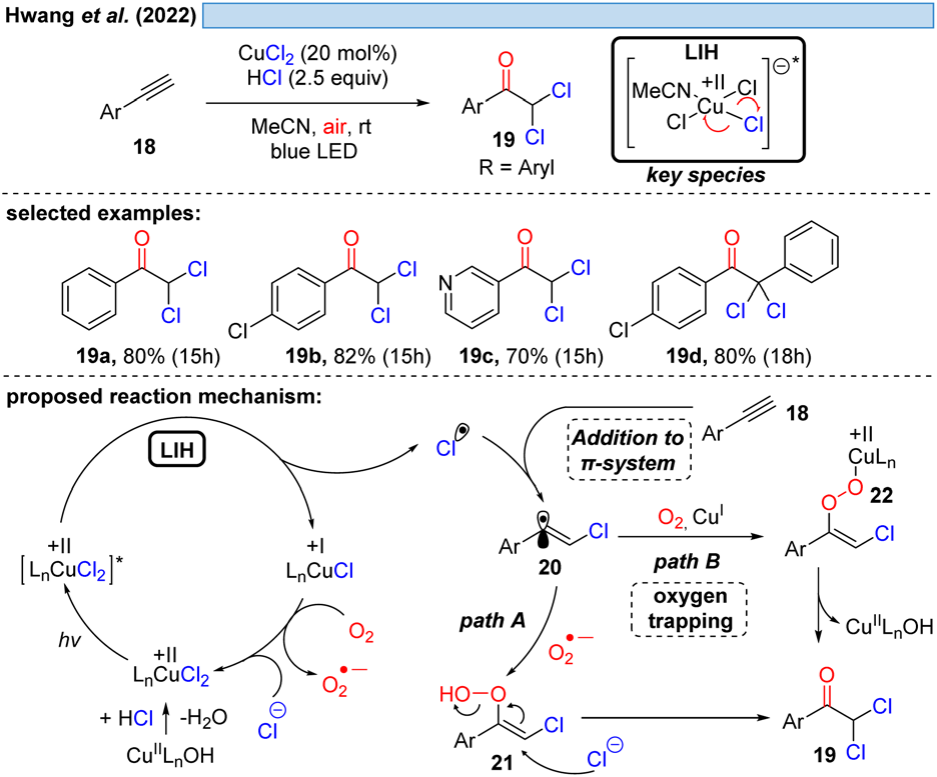
Synthesis of α-keto-dichlorination of arylalkynes.

Consistent with this reasoning, in 2022 the group of Hwang reported the visible-light induced oxidative α-keto-dichlorination of arylalkynes 18 promoted by CuCl_2_ at room temperature (Scheme 6).^26^ Also in this case, the reoxidation of Cu(i) by oxygen with concurrent formation of O_2_˙^−^*via* path A is proposed, which should recombine with 20 to ultimately give rise to product 19. O_2_˙^−^ could be indeed detected by EPR, nevertheless, its trapping with 20 would call for the recombination of two short-lived species present in low concentration. An alternative is conceivable *via* path B, *i.e.* the direct trapping of 20 with oxygen followed by recombination of the resulting peroxy radical with Cu(i) to intermediate 22 thereby regenerating Cu(ii) (*cf.*Scheme 11).

**Scheme 7:**
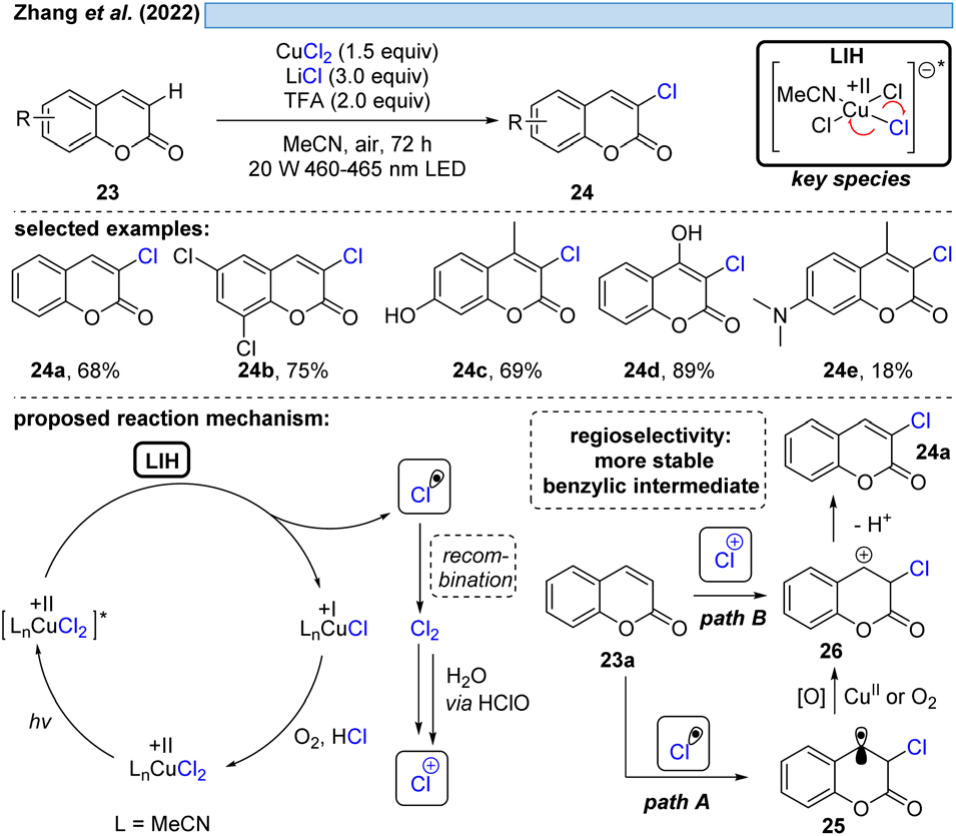
Regioselective chlorination of coumarins.

The group of Zhang described a synthetic procedure for the efficient regioselective chlorination of coumarins 23 (Scheme 7).^27^ Again, LIH of CuCl_2_ delivered chlorine radicals. Direct regioselective addition of the Cl radical to the 3-position of coumarin 23a would give rise to a stable benzylic radical 25 (path A). SET oxidation of the benzylic radical by either oxygen or CuCl_2_ followed by deprotonation leads to the chlorinated product 24a. Alternatively, the authors assume the chlorine radicals could recombine to deliver Cl_2_. A follow-up reaction with water delivers hypochlorite as a positive chlorine source. Selective electrophilic addition of Cl^+^ to coumarin's double bond at the 3-position (path B) and subsequent deprotonation could afford the chlorinated products 24. Also this example underlined that the reoxidation of Cu(i) to Cu(ii) by oxygen is not efficient, given that again CuCl_2_ needs to be employed in overstoichiometric, rather than in catalytic amounts.

Inspired by previous work on LIH of Cu(ii)-intermediates, Cai and co-workers developed a catalytic copper-catalyzed protocol for the synthesis of α-chloroketones from aromatic alkenes including electron-deficient olefins under an oxygen atmosphere, employing MgCl_2_ as a chlorine source in the presence of trifluoroacetic acid (TFA).^28^ Mechanistic studies, such as radical trapping experiments, showed that the peroxo Cu(ii)-species is the key intermediate and hydroperoxyl (HOO˙) and chlorine (Cl˙) radicals are generated. The following path is proposed: LIH delivers a chlorine radical, which rapidly adds to olefin 27 to give the benzylic C-centered radical 32. The subsequent reaction of intermediate 32 with oxygen affords O-centered radical 33, which can bind to 31 to afford the peroxo Cu(ii)-complex 34 (*cf.*Scheme 11), followed by the formation of the intermediate 35. Finally, the product 28 is obtained through the elimination of water from 35.

#### 1.1.2 LIH of CuCl_2_ – chlorine radicals as HAT catalysts

**Scheme 8:**
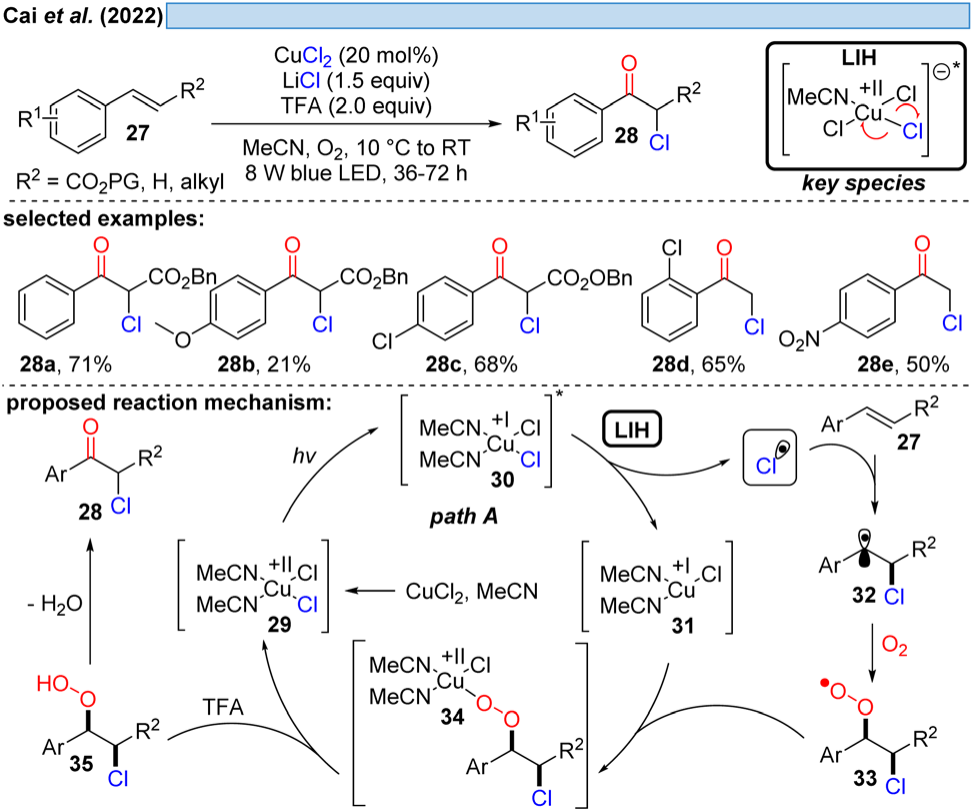
α-Chloroketonation of aromatic alkenes.

**Scheme 9:**
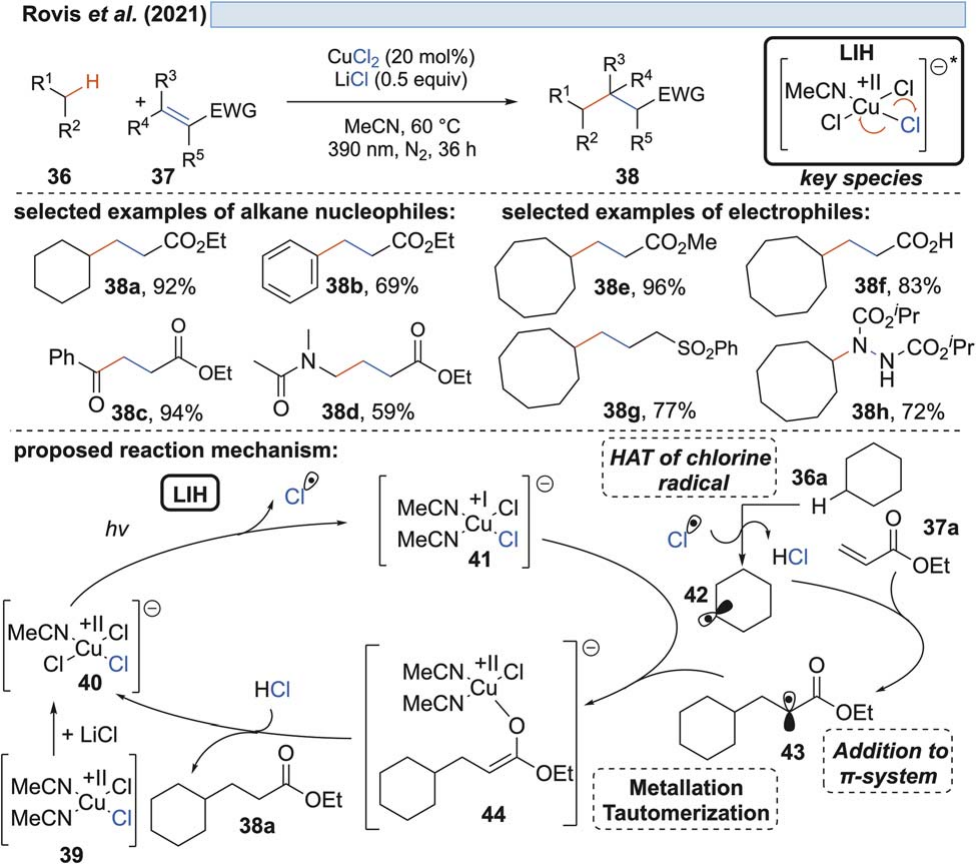
CuCl_2_-mediated activation of aliphatic feedstock chemicals.

Besides the addition to C–C-double bonds, the chlorine radicals generated by light-induced homolysis of Cu^II^Cl_2_ also prove to be excellent hydrogen atom transfer (HAT) reagents. Driven by the formation of a strong H–Cl bond (BDE = 103 kcal mol^−1^) the chlorine radicals so generated by Rovis and coworkers demonstrated the activation of C(sp^3^)–H bonds in alkanes 36 (Scheme 9).^29^ The resulting nucleophilic alkyl radicals can be coupled with electron-deficient olefins 37 to give rise to hydroalkylation products 38. Although a comparable high loading of CuCl_2_ (20 mol%) is employed, the overall process is nevertheless catalytic in Cu(ii) without the need for an external oxidant such as oxygen (*cf*. Schemes 7 and 8). The critical reoxidation to Cu(ii) is proposed to occur by the combination of radical 43 and Cu(i), *i.e.* representing the reversal of the known one-electron oxidation of enolates by Cu(ii).

**Scheme 10:**
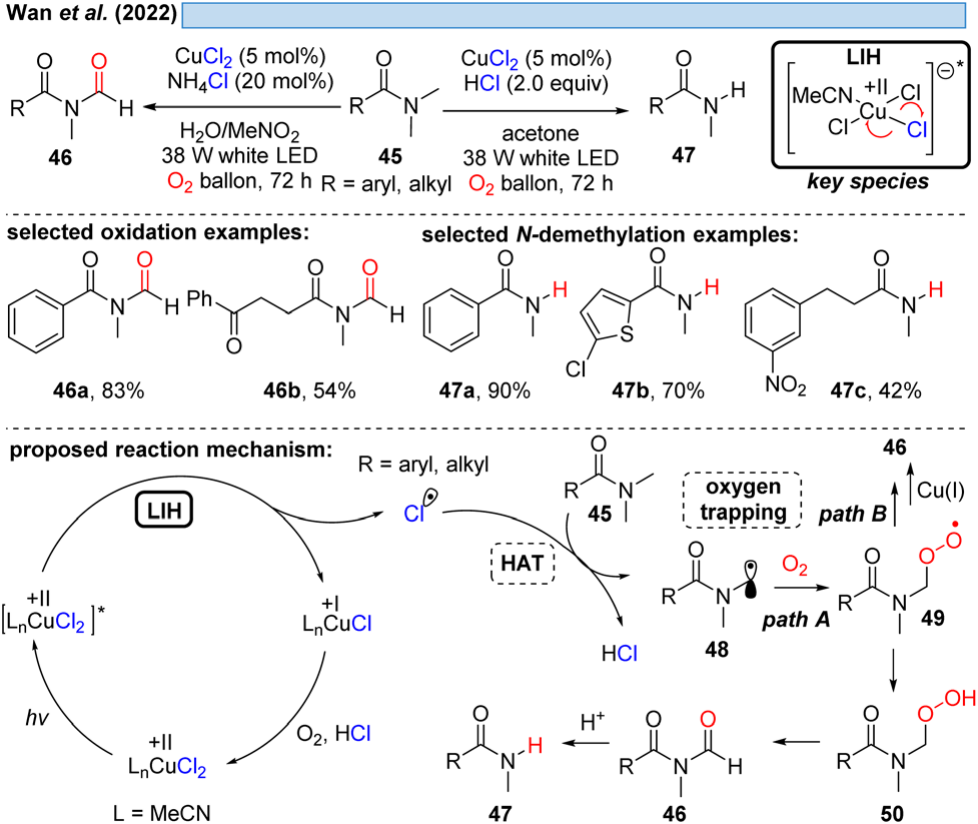
Photocatalytic functionalization of amine derivatives.

Likewise, α-amino radicals can be formed by H-abstraction from amine derivatives 45, including secondary and tertiary amines, sulfonamides, carbamates and α-ketoamides,^30^ which then can be oxidized by oxygen with concurrent regeneration of the Cu(ii) as discussed before (*cf.*Schemes 6–8). The ultimate outcome of this process is oxidation to either *N*-formyl derivatives 46 or – under more acidic conditions – the demethylated adducts 47 by the subsequent deformylation (Scheme 10). The critical reoxidation of Cu(i) to Cu(ii) is proposed by the authors to occur directly by oxygen, which would also call for an additional hydrogen abstraction step of peroxide 49 to 50. As discussed before, a plausible alternative even here would be the oxidation of Cu^I^ by peroxide 49 with the formal elimination of Cu^II^–OH to form the products.

### 1.2 LIH of copper(ii)–N_3_ – azide radicals

**Scheme 11:**
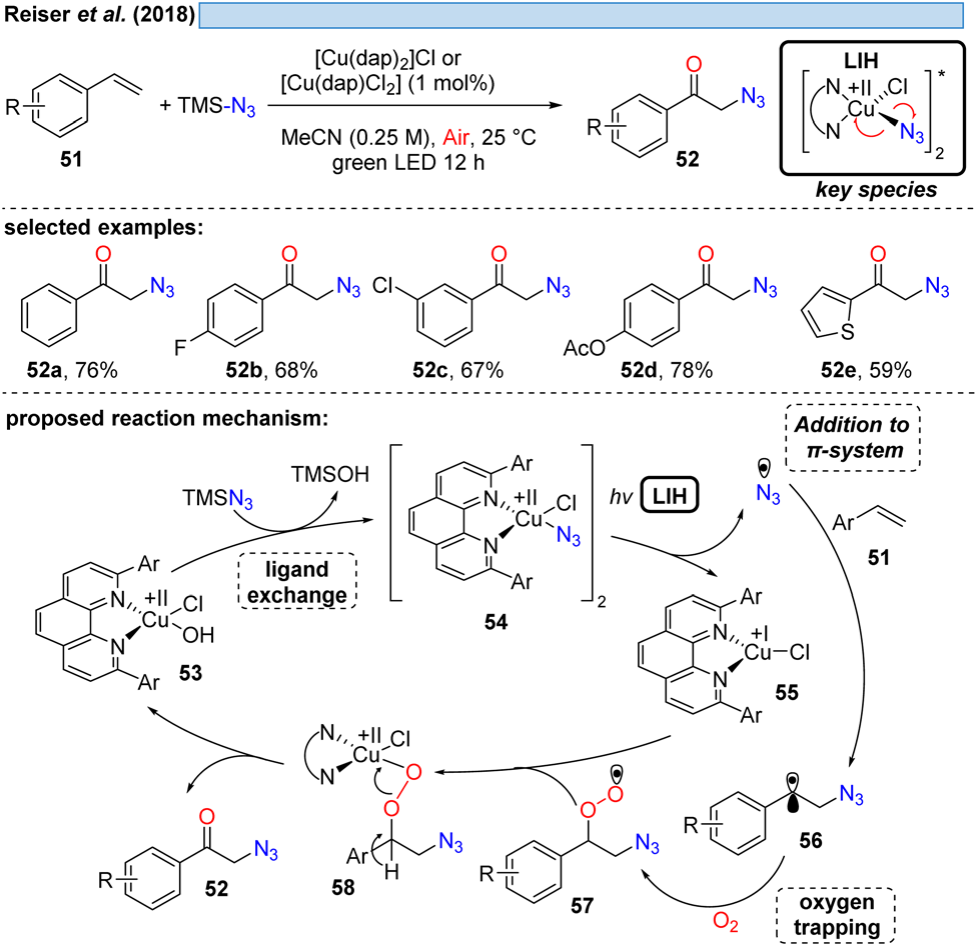
Copper(ii)-photocatalyzed oxo-azidation of styrenes.

The first example showing that complexes amenable to LIH can be generated *in situ* and thus allowing the employment of a Cu(ii)-complex in catalytic amounts was reported in 2018 (Scheme 11). Employing TMS–N_3_ as a stoichiometric azide source, azide/chloride substitution with [Cu^II^(dap)]X_2_ gave rise to the key Cu(ii)–azide intermediate 54.^31^ Upon LIH, the resulting azide radicals underwent addition to a broad range of vinyl arenes, followed by termination of the transformation with concurrent regeneration of the Cu(ii)-catalyst *via*57 and 58 along the lines that have been discussed before.

### 1.3 LIH of copper(ii)–CH_2_R – benzyl radicals

**Scheme 12:**
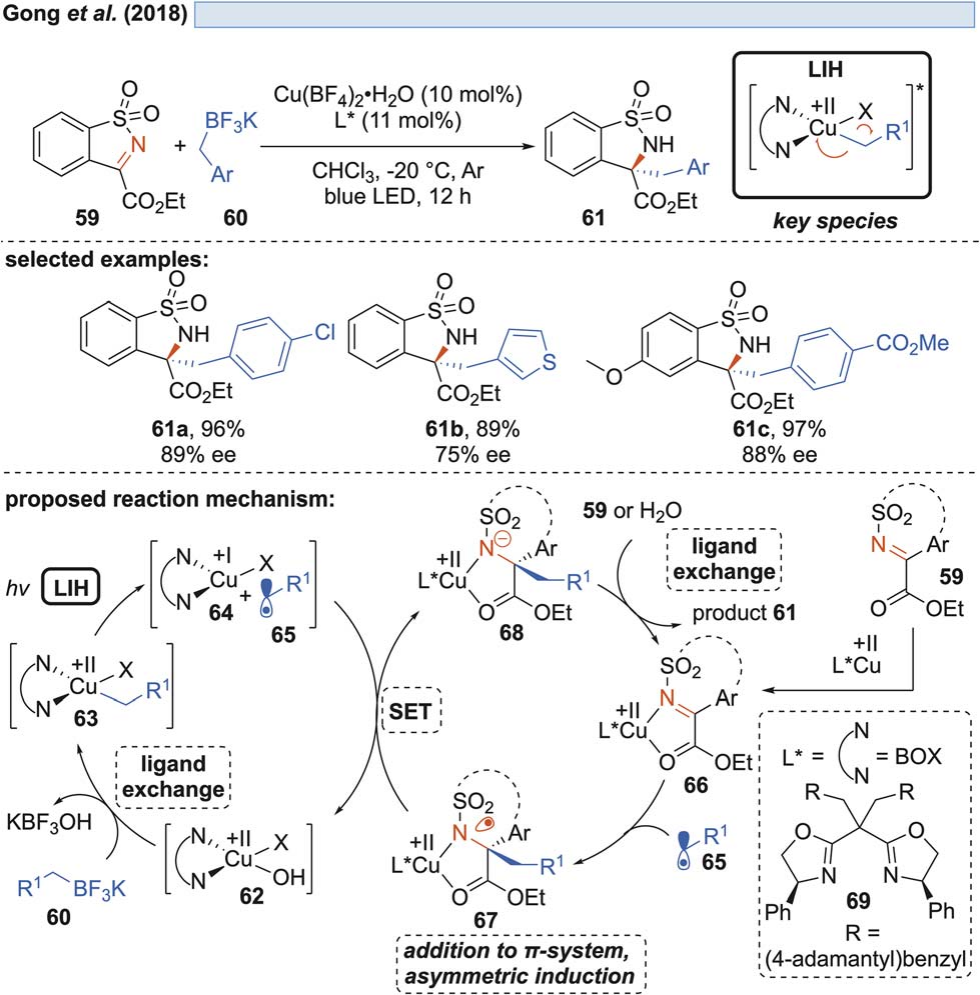
Enantioselective alkylation of imines.

Shortly thereafter, Gong and co-workers demonstrated that by another ligand exchange reaction, *i.e.* by the transmetalation of benzyl trifluoroborate salts, *in situ* formed copper(ii)-benzyl complexes 63 can undergo LIH to form benzyl radicals 65 (Scheme 12).^32^

The alternative generation of 65 by direct oxidation of the trifluoroborate (*E*_ox_ = +1.34 V *vs.* SCE)^32^ salts by Cu(ii) was ruled out in light of the insufficient reduction potential (Cu(ii) → Cu(i), *E*_red_ = +0.50 V *vs.* SCE).^20,21^ Radicals 65 were enantioselectively trapped by sulfonylimines 59, being activated by a chiral bis(oxazoline)–Cu(ii) complex Cu(ii) 66. Notably, the intermediate 67 formed after addition of 65 was found to undergo reduction by Cu(i), thus releasing the alkylated product 61 with concurrent regeneration of the Cu(ii)-catalyst 62 without the necessity of employing an external oxidant.

### 1.4 LIH of copper(ii)-enolates – enolate radicals

**Scheme 13:**
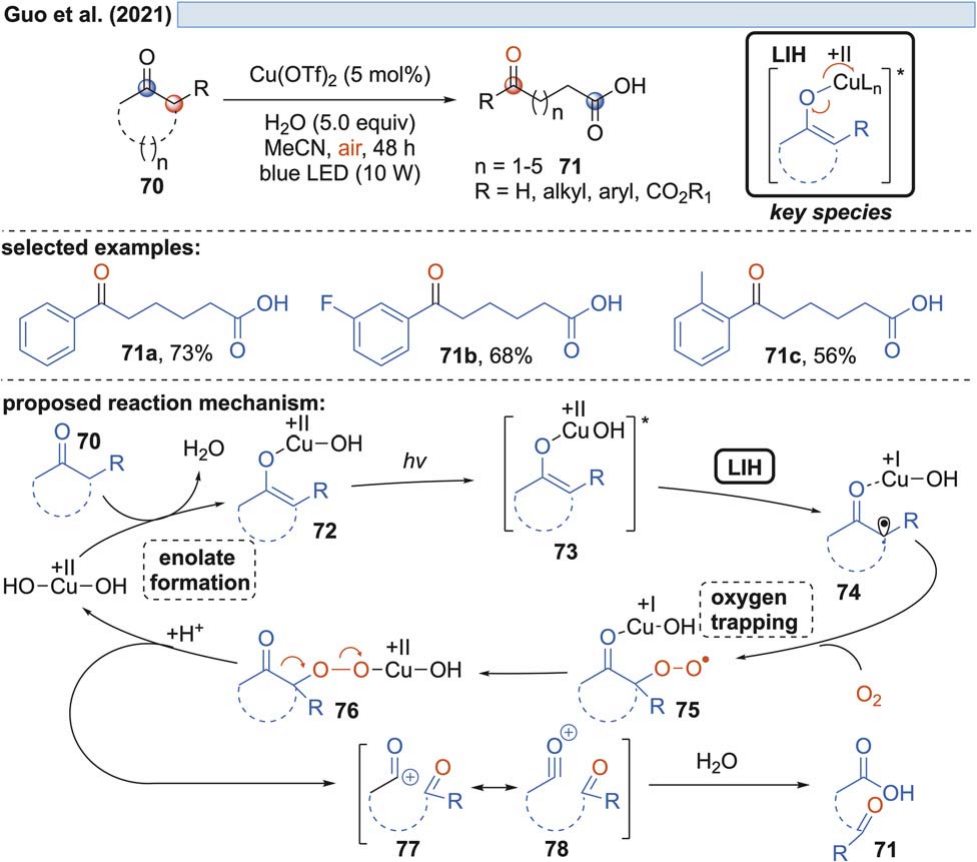
Aerobic oxidative cleavage of cycloalkanones.

Capitalizing on the known oxidation of enolates by Cu(ii) under thermal conditions,^33^ Guo and coworkers succeeded in the oxidative cleavage of α-phenyl-substituted cycloalkanones 70 to ketoacids 71. Operationally simple, a catalytic amount of Cu(OTf)_2_ in aqueous MeCN under blue light irradiation is sufficient to achieve the transformation (Scheme 13).^34^ Oxygen again is necessary to trap the enolate radical 65 formed by LIH of the excited copper-enolate 73, and in the absence of an α-hydrogen, a carbon elimination to the acylcation 77 occurs instead, which gives rise to 71 after the addition of water.

**Scheme 14:**
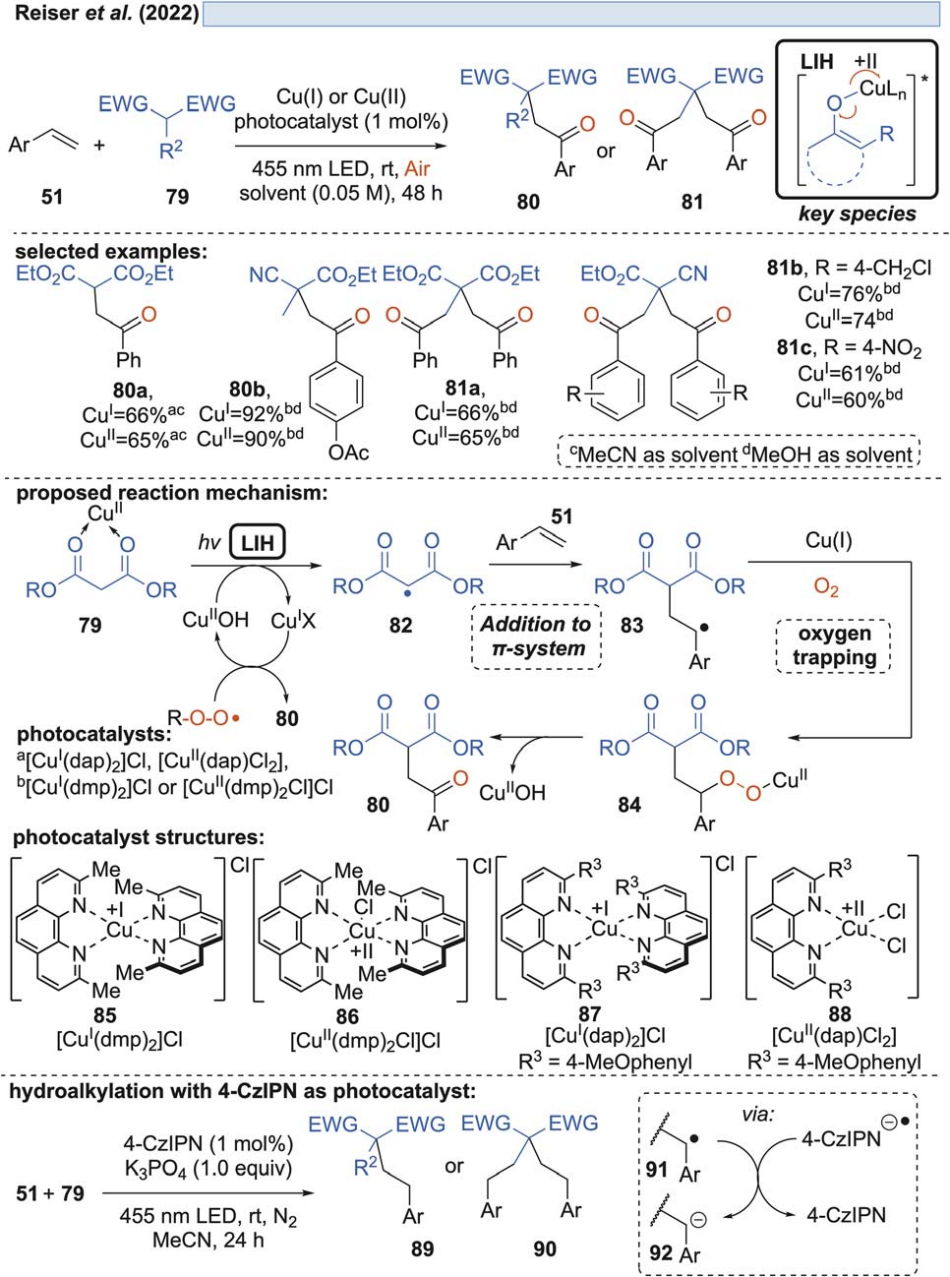
Cu(ii)-photocatalyzed oxo-alkylation of vinylarenes.

Likewise, radicals derived from 1,3-dicarbonyl compounds 79 can be generated *via* copper(ii)-induced LIH (Scheme 14) and utilized in a productive way to give rise to oxoalkylated vinyl arenes 80/81.^35^ Again, oxygen is necessary as a terminal oxidant to regenerate the Cu(ii)-photocatalyst presumably *via*84, *i.e.* the formation of a peroxo radical that is trapped by Cu(i). The importance of this step is underlined by the fact that if 1,2,3,5-tetrakis(carbazol-9-yl)-4,6-dicyanobenzene (4-CzIPN) is used instead of Cu(ii), the reduced photocatalyst 4-CzIPN˙^−^ is capable of transforming 91 to its corresponding anion 92 resulting in hydroalkylated products 89/90. Thus, a divergent photocatalytic transformation becomes possible, and notably, the oxoalkylation could not be achieved with 4-CzIPN and in turn, the hydroalkylation was not possible with Cu(ii).

### 1.5 LIH of copper(ii)–amine-complexes – amino radicals

**Scheme 15:**
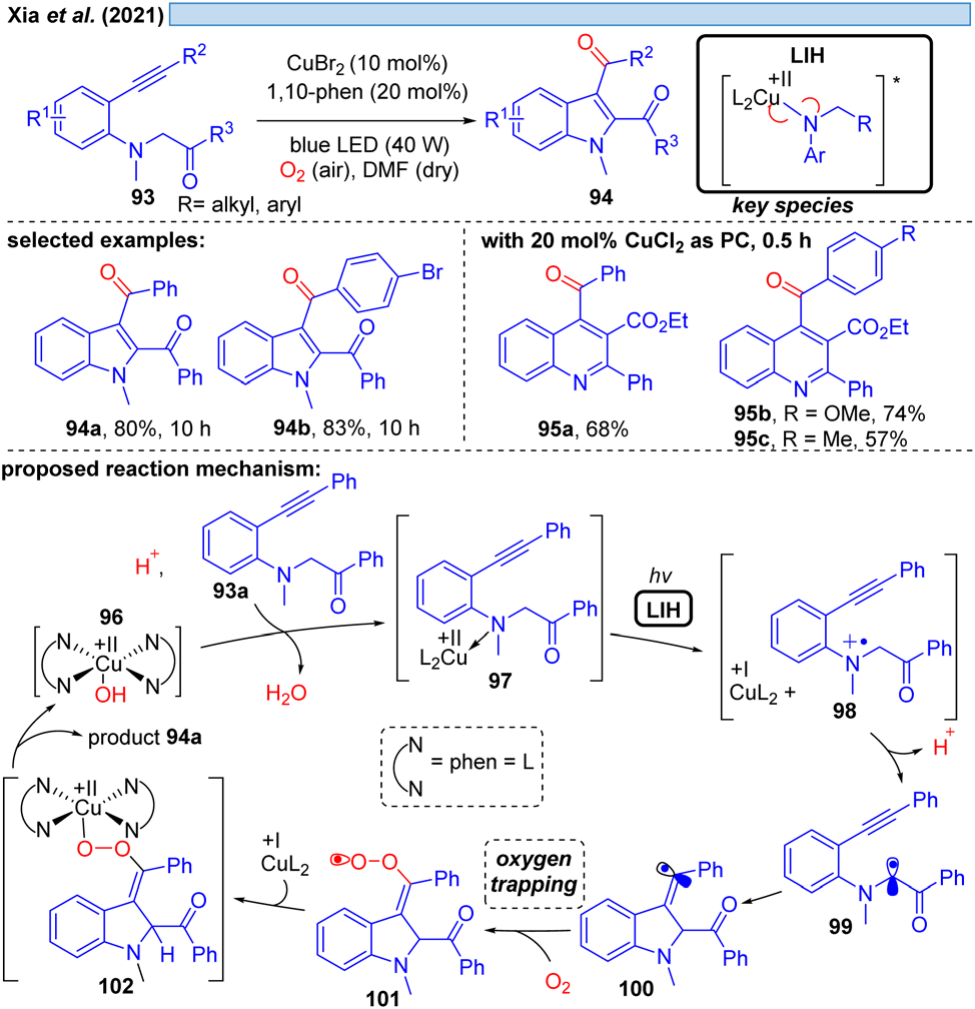
Cu(ii)-photocatalyzed oxidative cyclization reaction of aromatic amines.

Xia *et al.* disclosed in 2021 a Cu(ii)-photocatalyzed intramolecular oxidative cyclization reaction of substituted aromatic amines 93 and the C(sp^3^)–H bond adjacent to nitrogen with alkynes or alkenes, affording multi-substituted indoles 94 and quinolines 95 with dioxygen as a terminal oxidant (Scheme 15).^36^ The authors propose the initial oxidation of 93a to the radical cation 98 by Cu(ii). The formation of a N*-*coordinated species with Cu(ii) of type 97 was suggested based on UV studies, and thus it appears plausible that LIH of the Cu(ii)–N bond occurs, affording after proton loss α-amino radical 99, which rapidly undergoes cyclization to carbon-centered radical 100. Trapping of the latter by oxygen along with reoxidation of Cu(i) eventually leads to product 94a, which we would like to suggest could proceed *via*101 and 102 as discussed before for the oxoazidation (Scheme 11) or oxoalkylation (Scheme 14).

**Scheme 16:**
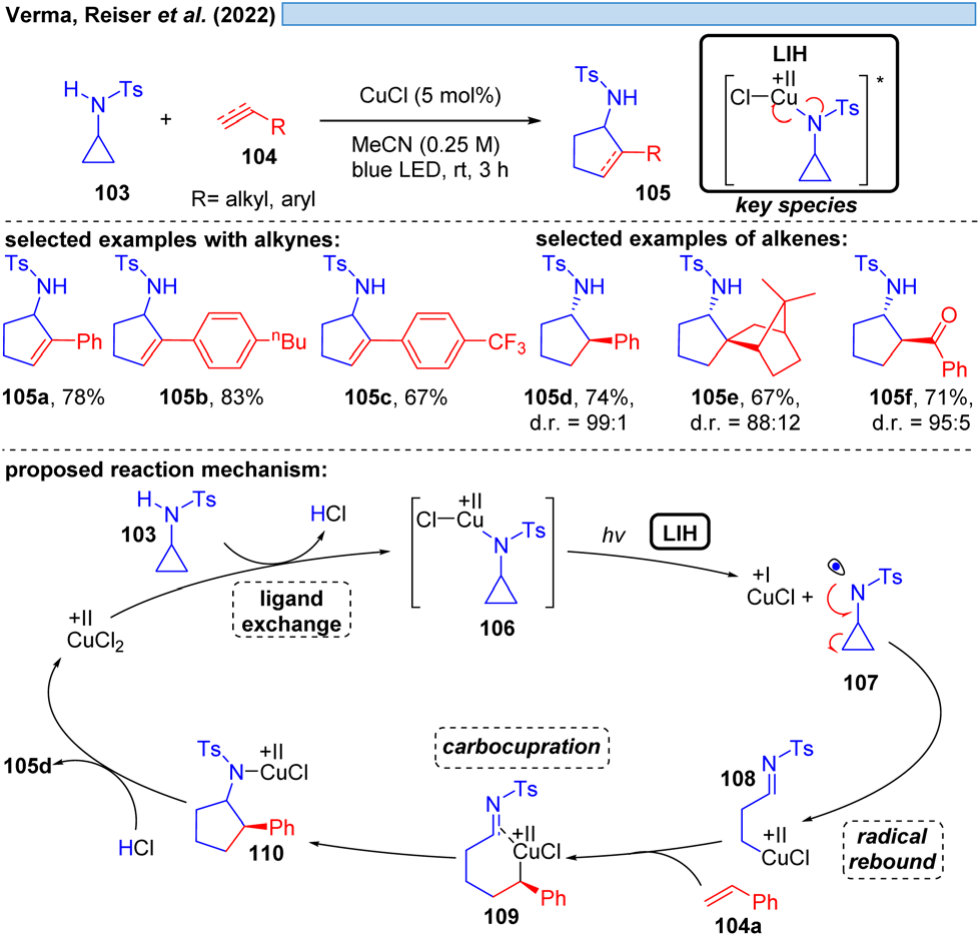
Visible-light-accelerated copper-catalyzed [3 + 2] cycloaddition of *N*-tosylcyclopropylamines with alkynes/alkenes.

Likewise, N-centered radicals can be formed from Cu(ii)–amide complexes *via* LIH (Scheme 16).^37^ It was recognized that an arylsulfonyl group on nitrogen is decisive for the success of the transformation, providing sufficient stabilization for the resulting nitrogen in contrasting *N*-benzoyl protecting groups. Moreover, an *N*-cyclopropyl substituent ensured the rapid transformation *via* an irreversible ring-opening of the cyclopropyl-*N*-radical 107 upon its inception. Thus, [3 + 2] cycloaddition of *N*-tosylcyclopropane 103 with different alkynes and alkenes 104 could be achieved giving rise to aminocyclopentanes and aminocyclopentenes 105 with high regio- and diastereoselectivity. The latter was attributed to the possibility of Cu(i) interacting with the radicals formed, *e.g.* in intermediates 108 or 109, contrasting related transformations that are initiated by Ru(bpz)_3_(BF_4_)^38^ or 4-CzIPN.^39^ Notably, no external oxidant is required for these transformations, which might raise the question of whether Cu(ii) has acted as a Lewis acid rather than as a photoredox catalyst or whether the reaction could be thermally initiated in the absence of the copper-catalyst. Control experiments however demonstrated that the reaction does not proceed in the dark or in the absence of copper, ruling out such alternate mechanistic pathways.

### 1.6 LIH of copper(ii)-carboxylates – carboxyl, alkyl and aryl radicals

**Scheme 17:**
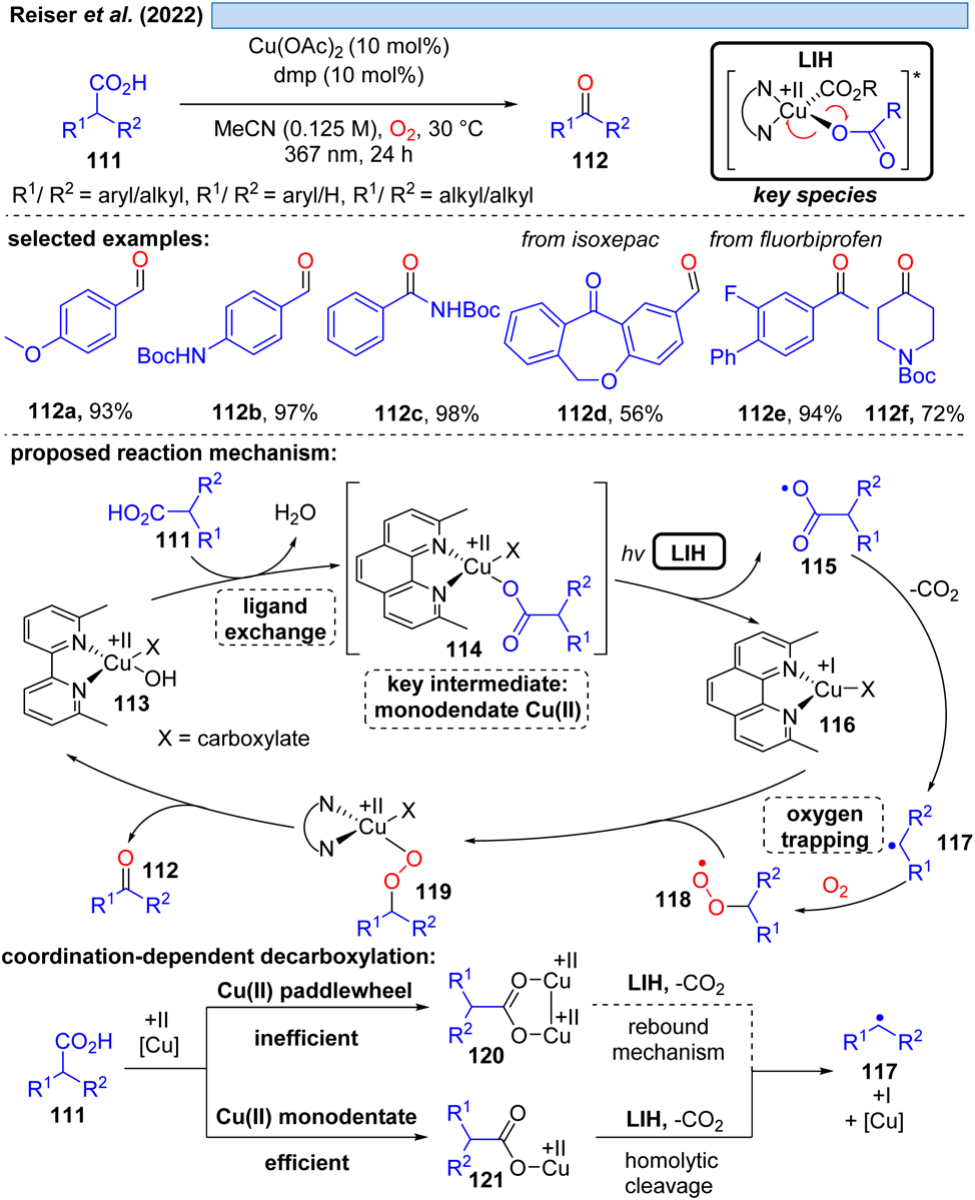
Decarboxylative oxygenation of carboxylic acids.

Although the decarboxylation of carboxylic acids under UV light irradiation in the presence of Cu(ii) salts has been known for a long time,^40^ it was not exploited for synthetic purposes. This seems to be surprising at first glance, given the potential to generate alkyl and aryl radicals from readily available feedstock, which should especially hold promise in converting renewable resources to value-added building blocks. However, the major challenge in this scenario apparently is to achieve catalytic turnover, *i.e.* after the oxidation of the carboxylate to turn back the resulting Cu(i) to Cu(ii). A possible explanation could be the high reactivity of the generated radicals against oxidants, most notably against oxygen. This rationale is corroborated by a recent study^41^ on the photocatalytic decarboxylation of phenylacetic or secondary carboxylic acids 111 in which a catalytic Cu(ii)/Cu(i)-cycle was achieved in the presence of oxygen as a terminal oxidant (Scheme 17). However, the radicals 117 generated upon decarboxylation could only be trapped by oxygen itself, all attempts towards their interception by other radicalophiles, notably by alkenes as was successful with malonyl radicals (*cf.*Scheme 14), failed. This study also revealed that the monodentate coordination, *i.e.*114/120, of a carboxylate to copper(ii) as opposed to the commonly encountered bidentate paddlewheel type 121 is greatly advantageous for achieving LIH.

**Scheme 18:**
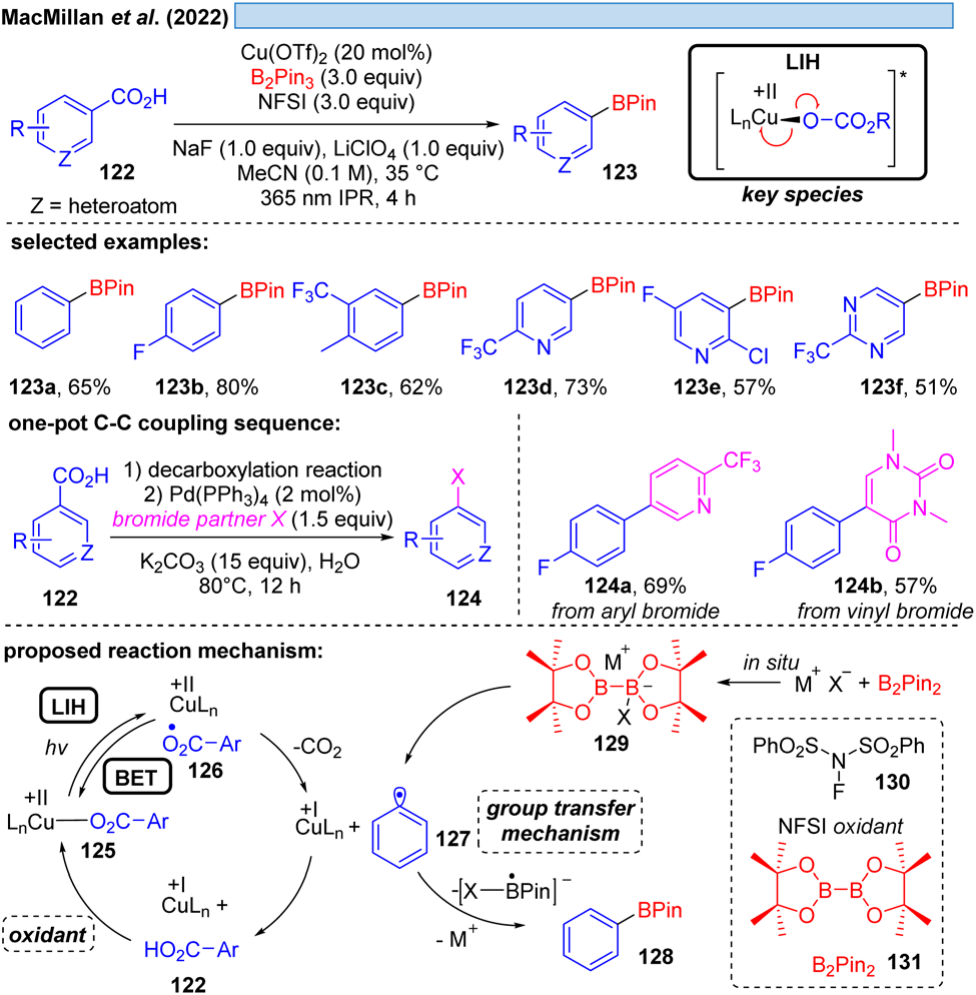
Decarboxylative borylation of (hetero)arylcarboxylic acids.

MacMillan and co-workers offered an alternative cooxidant that also allowed catalytic turnover in copper(ii). The decarboxylative borylation of (hetero)arylcarboxylic acids 122 under high intensity 365 nm light (Scheme 18) was achieved with the combination of Cu(OTf)_2_ (20 mol%)/*N*-fluorobenzenesulfonimide 130 (NFSI, 3.0 equiv.).^42^ The latter was able to regenerate the Cu(ii)-carboxylate 125 from Cu(i) and carboxylic acid 122 to again participate in the decarboxylative light-induced homolysis. This protocol was further compatible with palladium catalysis, allowing the subsequent one-pot couplings in Suzuki–Miyaura arylation, vinylation, alkylation or coupling of heteroaryl-boronic esters, picking up the synthetic utility of the boronic ester products initially formed.

**Scheme 19:**
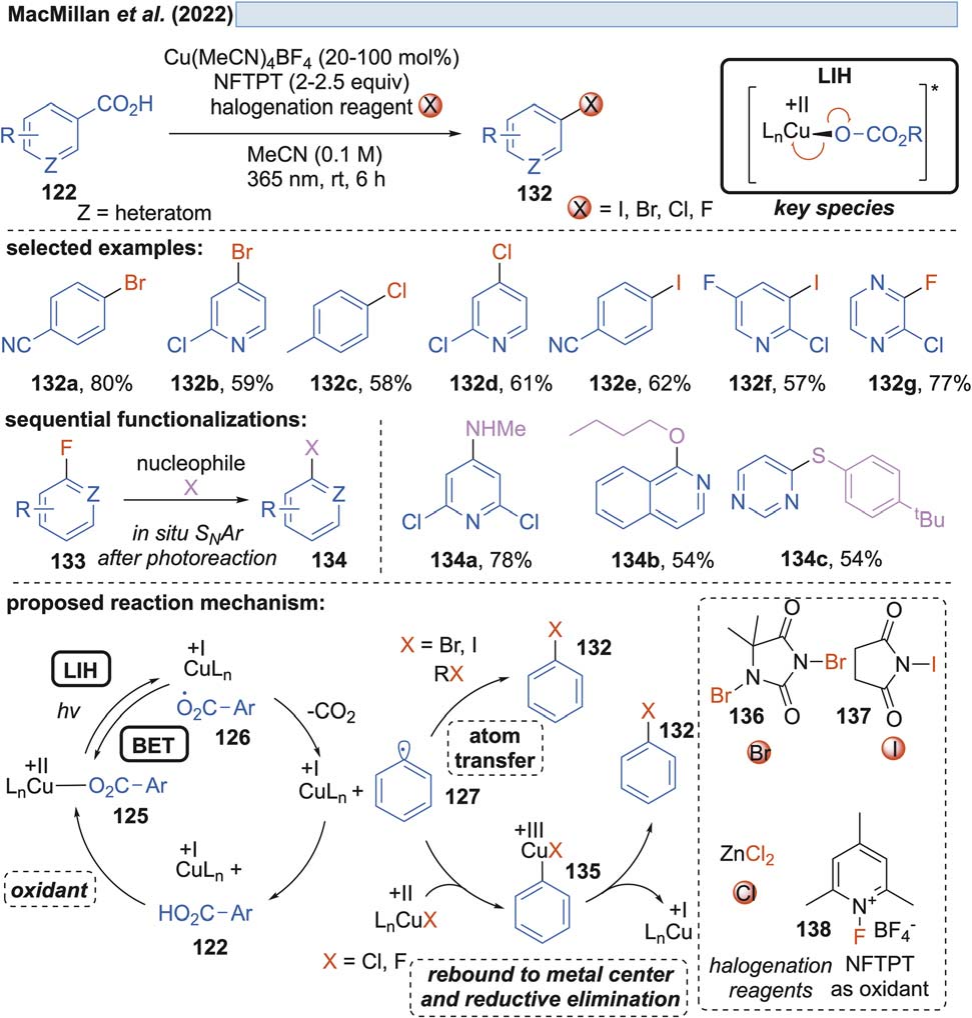
Decarboxylative halogenation of (hetero)arylcarboxylic acids.

**Scheme 20:**
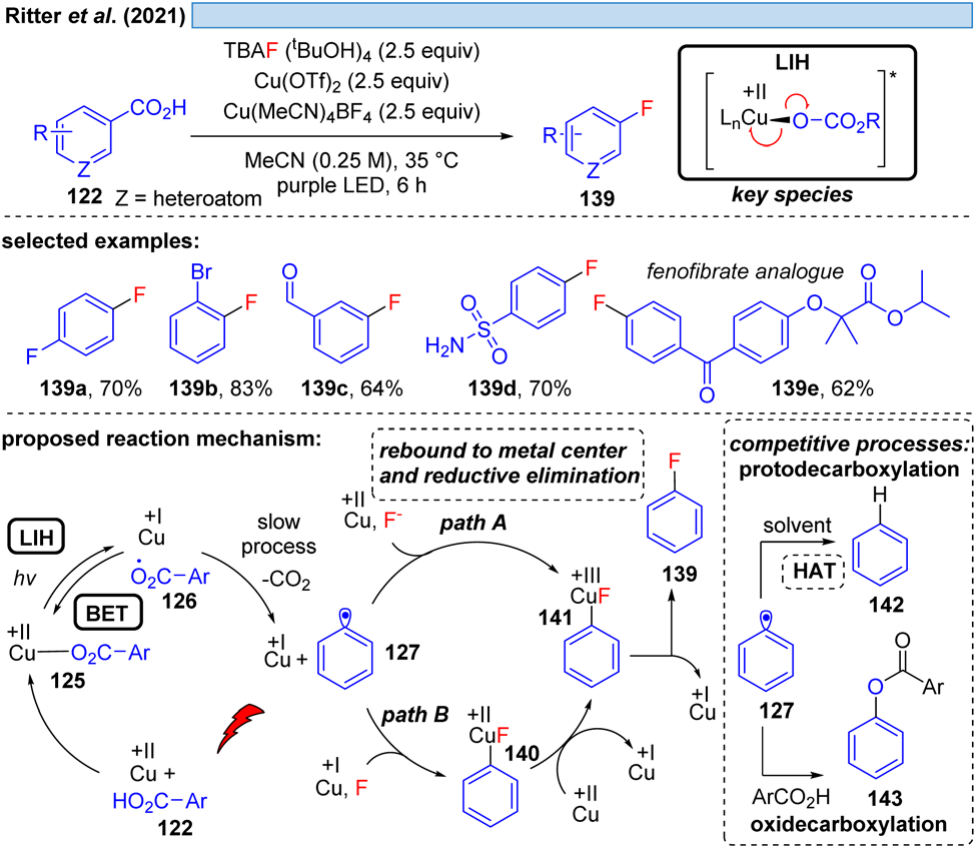
Cu(ii)-photocatalyzed decarboxylative fluorination of benzoic acids.

Along these lines, the decarboxylative halogenation of (hetero)aryl carboxylic acids became possible as well (Scheme 19).^43^ As the terminal oxidant 1-fluoro-2,4,6-trimethylpyridinium tetrafluoroborate (NFTPT) (138) was identified, which ultimately achieves the reoxidation of Cu(i) to Cu(ii) and thus allows the employment of substoichiometric amounts of Cu(ii). Two different pathways achieving the introduction of the halide nucleophile were considered. For bromination and iodination, atom transfer (AT) through the reagents 1,3-dibromo-5,5-dimethylhydantoin (136) or NIS (*N*-iodosuccinimide) (137) generated the products 132. Fluorination and chlorination were achieved with NFTPT (138) or ZnCl_2_ as a halogenating agent, which is proposed to occur by a rebound mechanism *via* a Cu(iii)-intermediate 135. Nevertheless, the fluorination protocol still required a stoichiometric amount of the copper salt as already observed by Ritter and co-workers (*vide infra*, Scheme 20). It remains unclear why the catalytic cycle is not closed in this case.

Nevertheless, the photooxidation of carboxylic acids by Cu(ii) as an entry point for alkyl and aryl radicals is highly attractive, making even the (over)stoichiometric use of Cu(ii)-salts an acceptable compromise for the synthetic transformations that can be achieved this way. In a series of elegant studies, this approach has been realized for a number of transformations that are difficult to achieve otherwise. The Ritter group has shown that copper(ii)-benzoates can serve as a facile entry point for introducing fluoride mediated by TBAF·(^*t*^BuOH)_4_ as the fluorine source (Scheme 20).^44^ Notably, the reaction tolerates functional groups such as aldehydes that are sensitive to oxidation, and moreover can be applied in the derivatization of bioactive compounds. Cu(ii) is proposed to be involved in two steps, being overall reduced to Cu(i) in each of them which explains the necessity of employing a minimum of two equiv. of Cu(OTf)_2_ in this transformation. Initially, LIH of a copper(ii)-arylcarboxylate 125 leads after decarboxylation of 126 to an aryl radical 127 and Cu(i). The decarboxylation of aryl carboxyl radicals (*k* ≈ 10^6^ s^−1^)^45^ is about 1000 times slower than the decarboxylation of alkyl carboxyl radicals (*k* ≈ 10^9^ s^−1^),^46^ and therefore it is assumed that the LIH step is reversible. The aryl radical 127 is then converted to the arylcopper(iii)-species 141, either by direct trapping with Cu(ii) (path A) or by Cu(i) followed by a one electron oxidation by Cu(ii) (path B), but no matter which pathway is followed, both require the employment of another equivalent of Cu(ii).

**Scheme 21:**
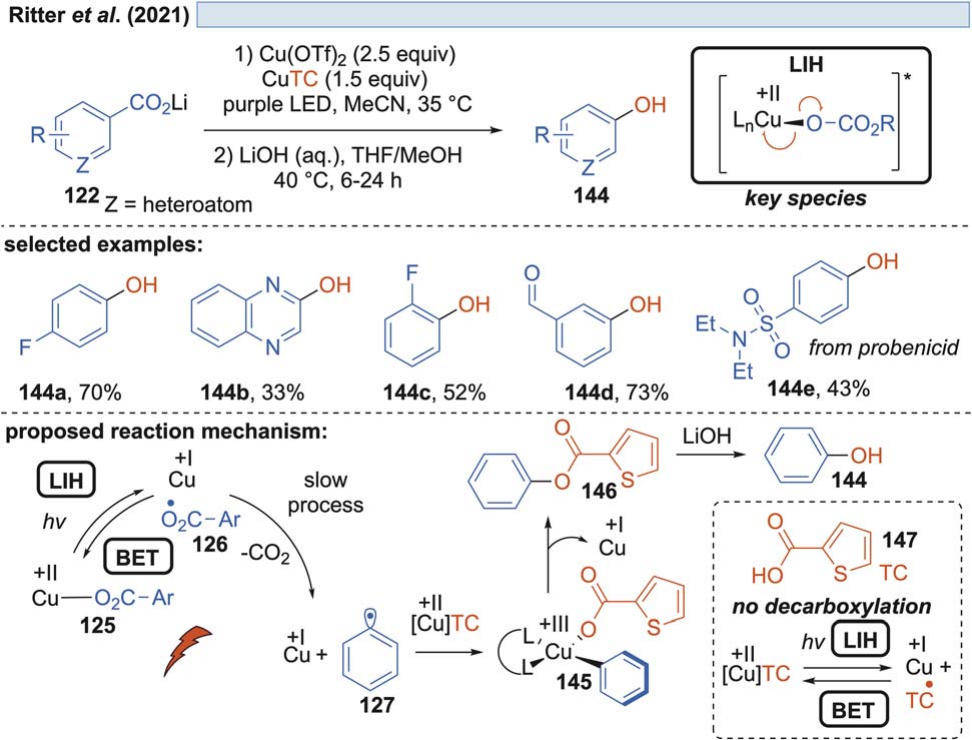
Decarboxylative hydroxylation of benzoic acids.

Besides fluoride, in a further study it was also realized that the carboxylate itself can act as the nucleophile, giving rise to benzoates 146, which upon hydrolysis yield valuable phenols 144 (Scheme 21). However, in this scenario, one equivalent of the carboxylic acid is sacrificed, allowing a maximum yield of only 50%. In a subsequent report by Ritter and coworkers, a solution to this problem was found by using thiophene-2-carboxylic acid 147 as the nucleophile.^47^ Realizing that although Cu(ii)TC might also undergo LIH to yield Cu(i) and the corresponding thiophene-2-carboxyl radical, the latter will not undergo decarboxylation. Therefore, thiophene-2-carboxylate can be regenerated most likely by back electron transfer (BET) with Cu(i) and thus Cu(ii)TC is available to allow trapping of the aryl radical 127 to the Cu(iii)-intermediate 145, which upon reductive elimination leads to the oxygenated arene 146 and Cu(i). Again, the catalytic cycle is broken at this point, which explains the necessity of employing an excess of Cu(ii) salts in the process.

**Scheme 22:**
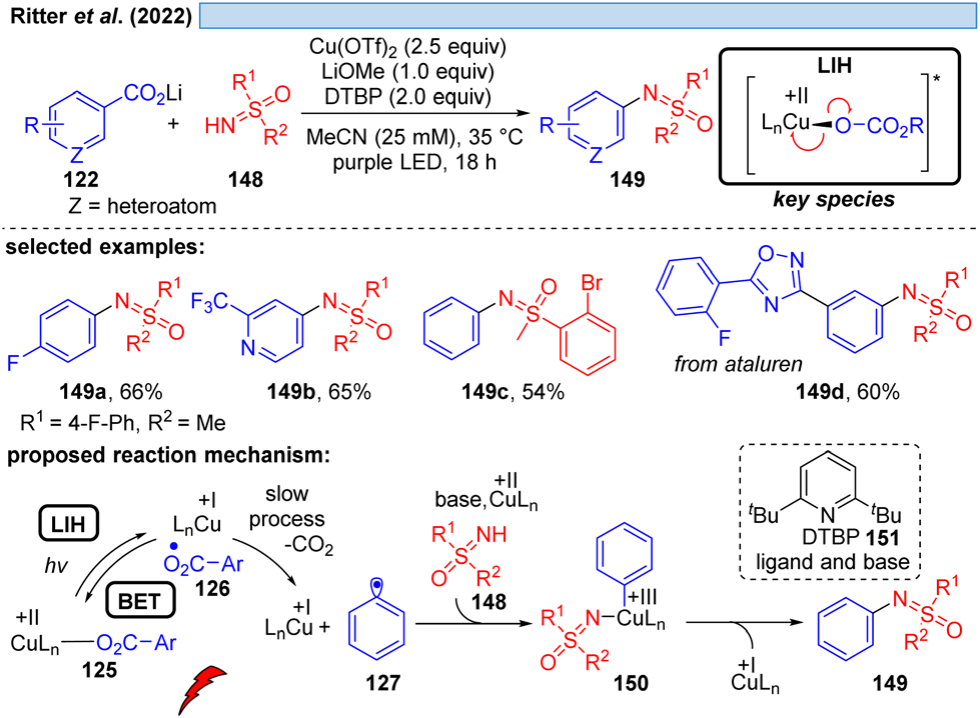
Decarboxylative sulfoximination of benzoic acids.

Likewise, the Ritter group developed a method for the decarboxylative sulfoximination of benzoic acids 122 (Scheme 22).^48^ It was found that lithium carboxylates with 2,6-di-*tert*-butylpyridine (DTBP) (151) and LiOMe as additives were required to achieve good reaction efficiency. The key step to enable this transformation was to overcome the formation of undesired sulfoximine-ligated Cu(ii)-species, as well as to suppress undesired oxide-carboxylation to phenols. The authors assume that the weak coordination of the DTBP ligand to Cu(ii) might favor C–N over C–O reductive elimination, or assist the formation of photoactive Cu(ii)-carboxylate species. The role of LiOMe remains unclear but it was assumed that it can decrease the concentration of free sulfoximines by forming weakly soluble sulfoximine lithium salts. Strong coordinating groups, or oxidizable groups, such as amines inhibited the transformation.

### 1.7 LIH of Cu(ii)-sulfoximines – N-centered sulfoximinyl radicals

**Scheme 23:**
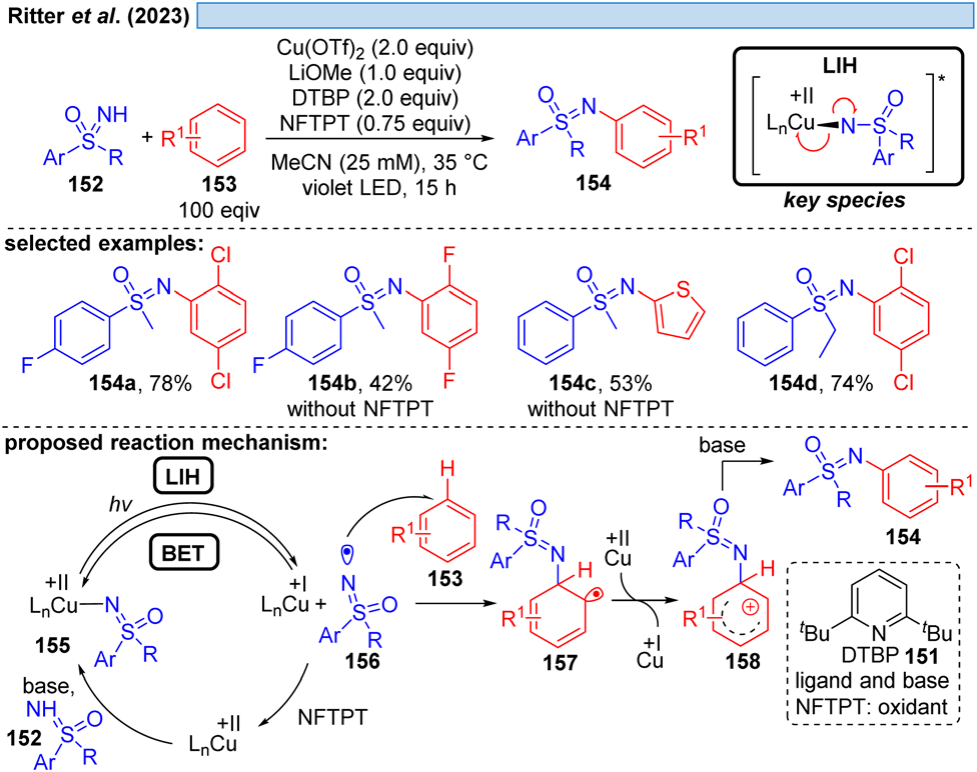
C–H sulfoximination of arenes.

Likewise, the Ritter group developed the C–H-sulfoximination protocol of arenes 153 (Scheme 23).^49^ The direct generation of sulfoximinyl radicals from NH-sulfoximines 152 (*e.g.* through HAT or SET) is challenging, because of the high oxidation potential (*E*_ox_ = +1.92 to +2.00 V *vs.* SCE)^50^ as well as the high bond dissociation energy (BDE, BDE_N–H_ = 104–106 kcal mol^−1^, by DFT calculation).^49^ However, as previously shown for *N*-tosylamines (*cf*. Scheme 16) N-centered radicals can also be accessed from NH-sulfoximines 152 using the principle of LIH. LiOMe served again as the best base to deprotonate 152, achieving Cu(ii)-sulfoximine complexes more efficiently. DTBP (151) was found to be a crucial ligand avoiding undesired BET through the stabilization of the Cu(ii)-sulfoximine complex 155. A broad range of electron neutral and rich arenes readily engaged in the transformation. Electron-deficient arenes were less reactive, resulting in the undesired HAT of sulfoximinyl radicals with unproductive consumption of Cu(ii) as a competing process. Thus, the addition of NFTPT as an oxidant was found to be beneficial for regenerating Cu(ii).

**Scheme 24:**
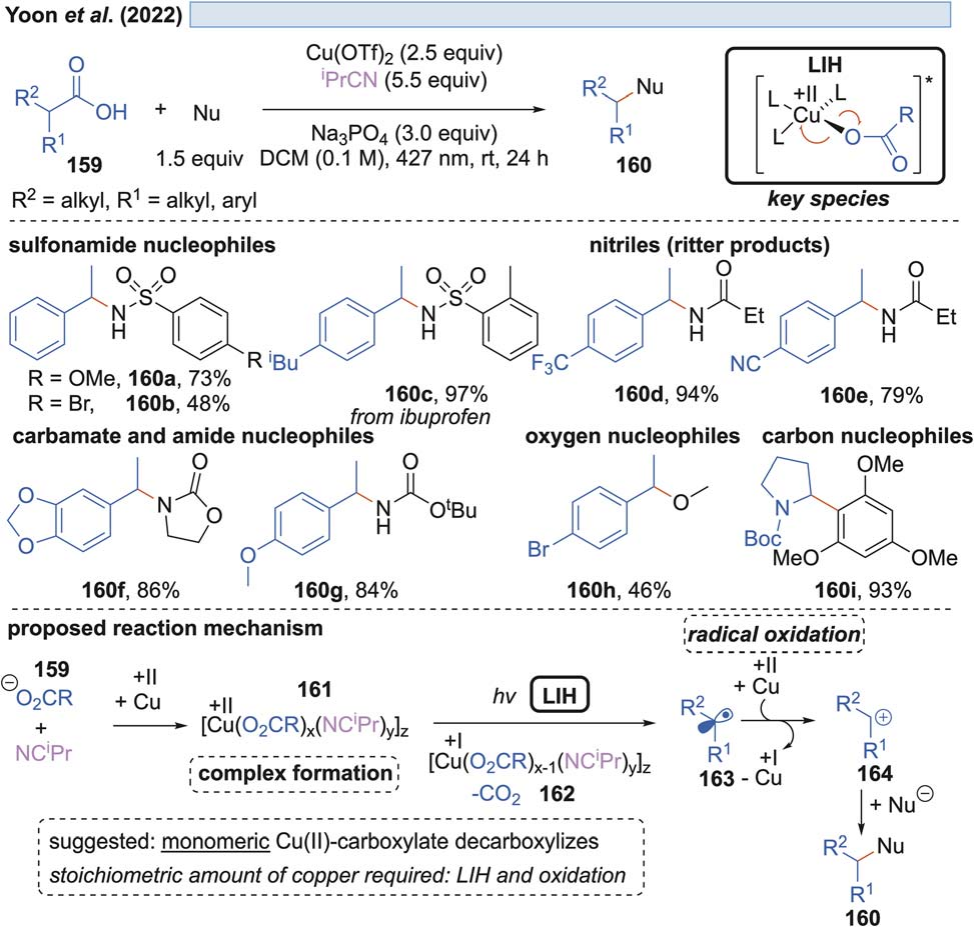
Decarboxylative cross-coupling reaction with different nucleophiles.

In the end of 2021 Yoon *et al.* reported a copper-mediated decarboxylative coupling of arylacetic and aliphatic carboxylic acids 159 and nucleophiles under irradiation at 427 nm (Scheme 24).^51^ The developed procedure has an exceptionally broad scope including nitrogen-, oxygen- and carbon-based nucleophiles. The copper(ii) salt fulfils two functions in the protocol, *i.e.* radical generation through the homolysis of Cu(ii)-carboxylate 161 and the subsequent oxidation of the benzylic radical 163 to the corresponding benzylic cation 164. Consequently, two equivalents of copper(ii) have to be applied, which was also the case in related work of this group.^52^ The application of ^i^PrCN as a ligand for copper was found to be essential to achieve the formation of catalytically active monomeric copper(ii)-carboxylates while dimeric copper(ii)-carboxylates are proposed to be photocatalytically inactive.

### 1.8 Outlook: transformations involving Cu^n^*/Cu^*n*−1^ transitions

**Scheme 25:**
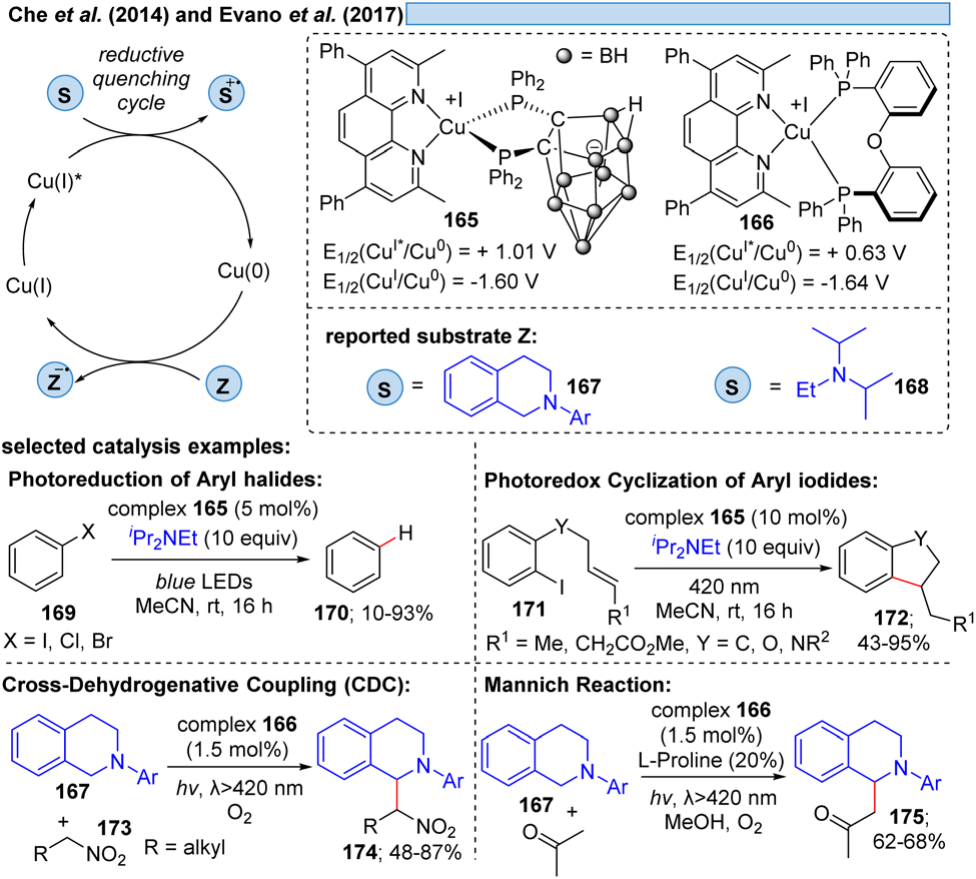
Heteroleptic complexes acting in the Cu(i)* → Cu(0) cycle.

Besides relying on Cu(ii)* to Cu(i) for the dissociative LMCT process, another possible copper transition for this transformation could be initiated by Cu(i)*→ Cu(0), however, only few examples exist for this reaction mode. Che and co-workers^11^ utilized the photostable zwitterionic copper(i)-complex 165 in a reductive quenching cycle involving a Cu(0) intermediate for the oxidation of amines (Scheme 25). In the same way, Evano *et al.*^12^ reported a heteroleptic copper-based photocatalyst 166. In these examples however, one can assume an outer-sphere mechanism for these oxidations.

**Scheme 26:**
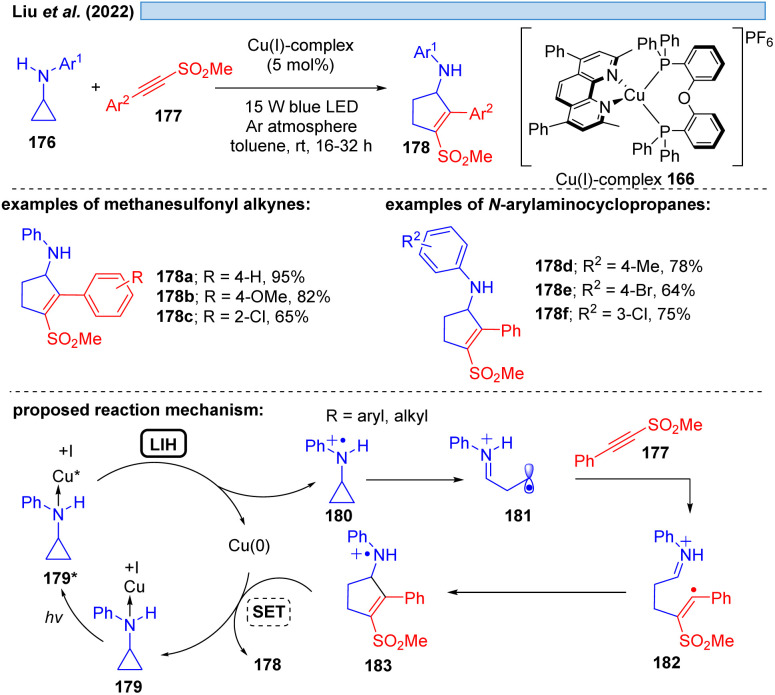
Copper-catalyzed [3 + 2] cycloaddition involving a Cu(i)*/Cu(0) cycle.

Evidence for LIH *via* a dissociative LMCT involving a Cu(i) → Cu(0) transition was put forward by Liu and co-workers^53^ for the [3 + 2]-cycloaddition of *N*-arylaminocyclopropanes 176 with alkynes 177, mirroring the transformation described by Verma, Reiser and coworkers^37^*via* Cu(ii)/Cu(i) cycles (*cf.*Scheme 16) (Scheme 26). Stern–Volmer studies indicate interaction of the excited Cu(i)-photocatalyst with *N*-arylaminocyclopropanes, thus, a precoordination of Cu(i) with aminocyclopropane 176 would be plausible. Subsequent SET from 179 to Cu(i)* affords cyclopropyl radical cation 180, which undergoes ring opening to deliver 181 and subsequently cycloaddition to yield the products 178 along with back electron transfer to regenerate the Cu(i)-photocatalyst.

**Scheme 27:**
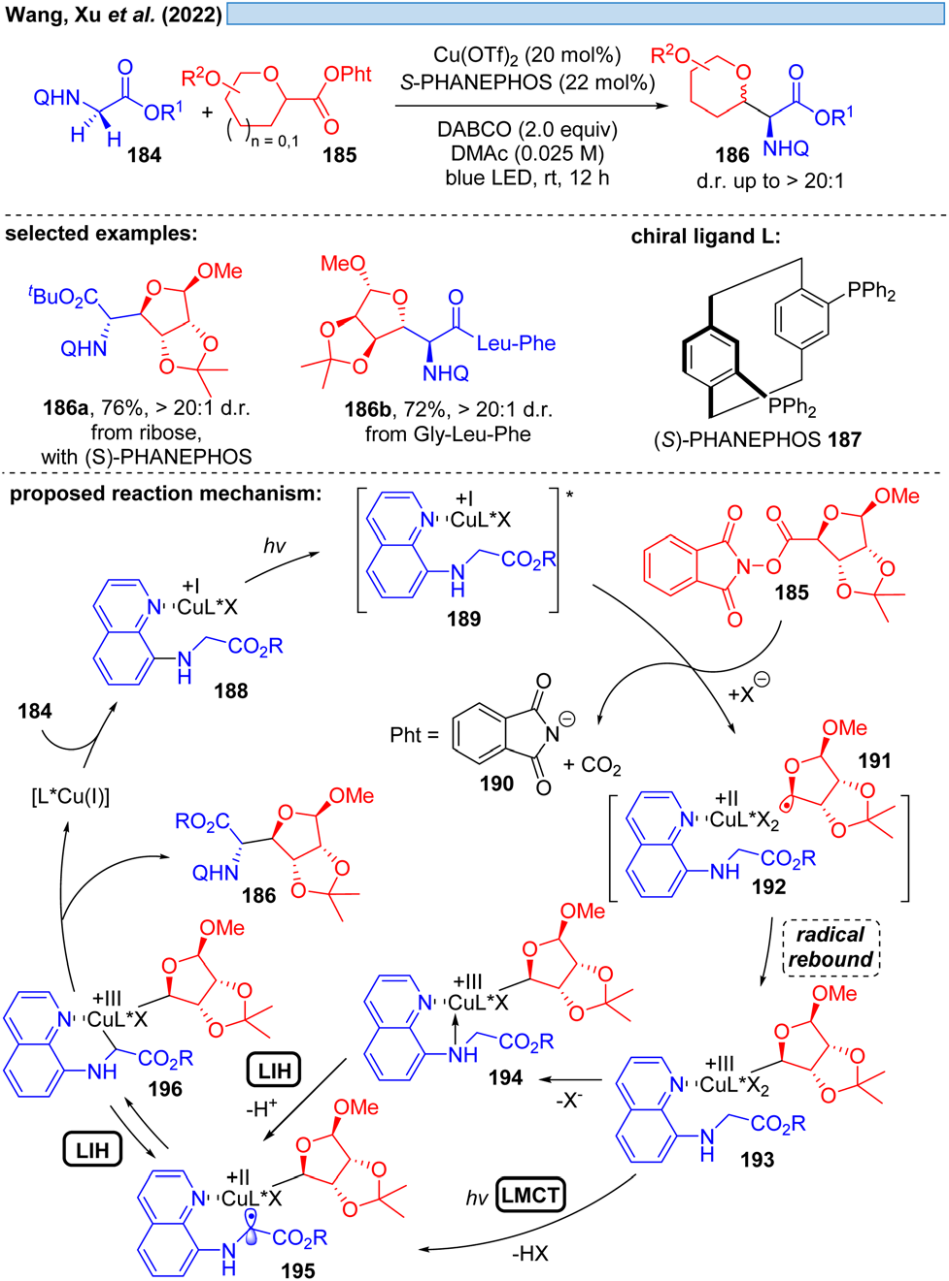
Copper-promoted C(sp^3^)–H glycosylation involving a Cu(iii)*/Cu(ii) cycle.

Another possible transition could be the transition of Cu(iii)* → Cu(ii). Cu(iii)-intermediates are generally elusive,^54^ but have been implicated in photocatalyzed processes *via* trapping of transient radicals by Cu(ii), which can be considered to be a persistent radical making this process efficient. However, reductive elimination resulting in Cu(i) is facile, accounting for the short-lived Cu(iii)-species. Nevertheless, in 2022, Xu and co-workers reported the visible-light promoted, stereoselective C(sp^3^)–H glycosylation for the synthesis of C-glycoamino acids and C-glycopeptides (Scheme 27).^55^ Assuming Cu(i) is generated *in situ* from Cu(ii), the catalytic cycle starts with established elemental steps, *i.e.* SET of the Cu(i)-substrate complex 189 to the second substrate 185 generates radical intermediate 191, which leads after rebound to Cu(ii) the critical Cu(iii)-intermediate 193. To achieve the coupling of the two organic moieties that ultimately give rise to the final product 186, the intermediate 196 appears to be plausible. The authors speculate that 196 can be formed from 193 by deprotonation and MLCT *via* a Cu(ii)-species of type 195. Evidence for the appearance of Cu(iii) is provided by UV-spectroscopy. Alternatively, a glycine moiety could undergo direct ligand exchange to 194 to afford 195, which could be in equilibrium *via* LIH with 196 to assume the most favorable geometry controlled by the chiral ligand 187 for the final reductive elimination.

## 2. Summary and prospects

**Scheme 28:**
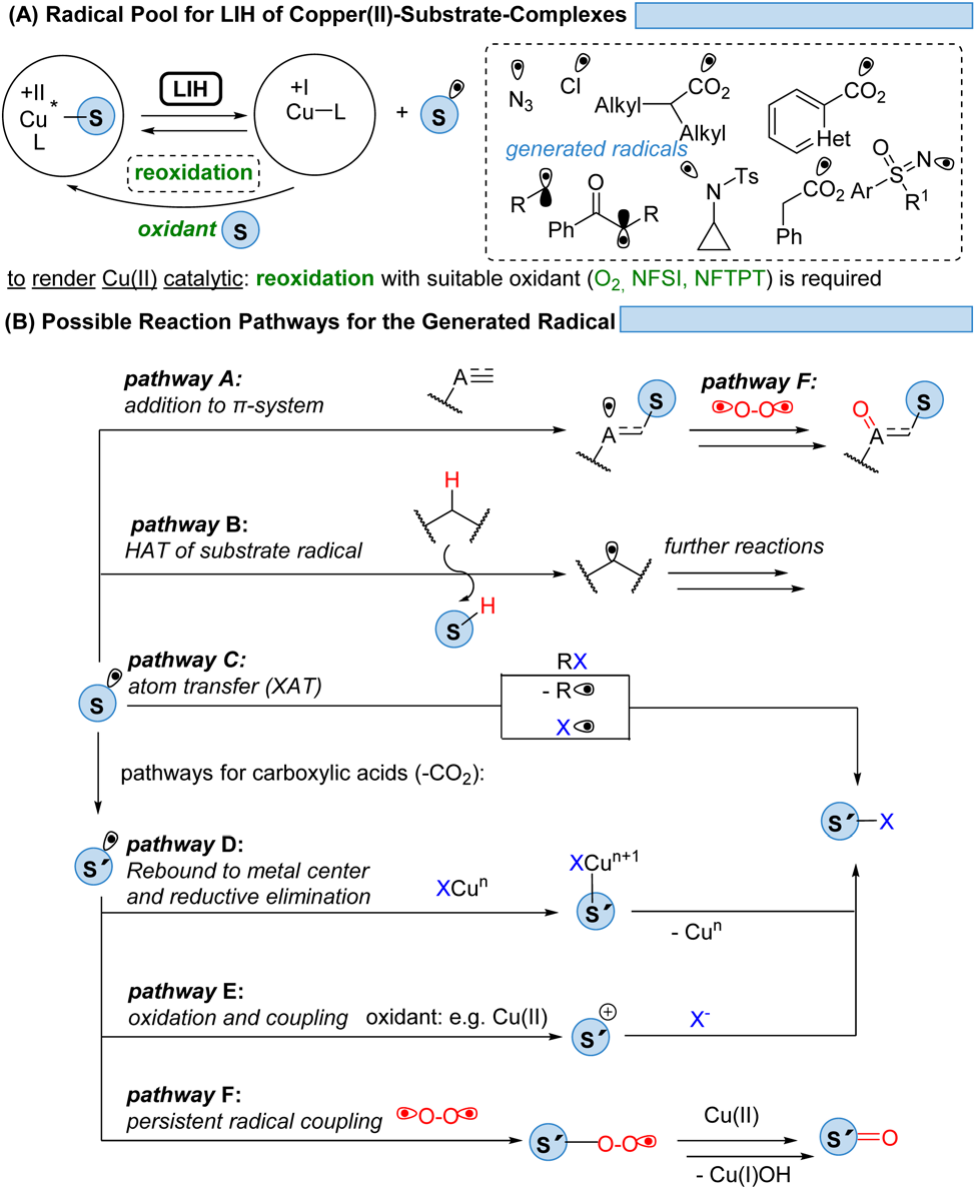
Prospects: achieving a platform for possible synthetic transformations.

Given the abundance of 3d-transition metals, their substitution for their precious 4d- and 5d-congeners is an active but challenging area of research. To overcome the problem of ultrashort excited-state lifetimes of 3d-metal complexes, the precoordination of substrates which undergo light-induced homolysis (LIH, which in most cases is dissociative LMCT) appears to be a promising concept (Scheme 28A). Spectroscopic evidence has been provided that such homolysis events are ultra-fast (<1 ps), thus pushing back on the relevance of excited-state lifetimes for radical generation by SET. Given the versatile coordination chemistry of Cu(ii), copper(ii)-complexes with azide, amine, sulfoximine, carboxylate, enolate, or alkyl substituents have been proven so far to be suitable substrates for LIH.

The mild conditions under which radicals S˙ can be generated this way in combination with the possibility of the copper catalyst interacting with and thus stabilizing the intermediates subsequently formed within a catalytic cycle have resulted in the development of distinctively different reaction pathways (Scheme 28B). Addition to π-systems (pathway A) has been demonstrated as the starting point for ATRA or ATRA-like processes. Hydrogen atom abstraction (HAT, pathway B) has proven to be a powerful tool for the functionalization of feedstock hydrocarbons. The radical S˙ might undergo a rapid fragmentation to a new radical S′˙ as it is best known for carboxyl radicals that extrude CO_2_ to give rise to alkyl or aryl radicals. From here, manifold atom transfer reactions (XAT) have been shown to become possible (pathway C) which might be preceded by the rebound of S′˙ to Cu(i) or Cu(ii) (pathway D). In the presence of a suitable oxidant, often achieved by employing additional equivalents of Cu(ii), the oxidation of S′˙ to the corresponding cation S′^+^ sets the stage for the coupling with nucleophiles (pathway E). Another possibility is a radical–radical coupling between the transient radical S˙ or S′˙ and a persistent radical, the best known being molecular oxygen, which gives rise to ketones or aldehydes (pathway F).

A crucial aspect of making the transformations discussed catalytic is the required reoxidation of Cu(i) to Cu(ii), which is in most cases only achieved by oxygen, thus resulting in the concurrent oxidation of the final product.

In general, a deeper understanding of the reactivity of substrates amenable to LIH is required. More fundamental investigations using combinations of photophysical methods as well as computational studies need to be carried out to identify possible substrates and synthetic transformations that can be addressed. Copper(ii) is not just from an economical and ecological point of view an attractive metal for photocatalytic transformations *via* LIH but also provides the possibility of copper(ii) interacting with and stabilizing radical intermediates through an inner-sphere mechanism which will open up new avenues and opportunities in the future, *e.g.* in asymmetric catalysis.

## Author contributions

Alexander Reichle and Oliver Reiser jointly wrote the manuscript.

## Conflicts of interest

There are no conflicts to declare.

## Supplementary Material
